# Skeletal Muscle and Circulating microRNAs Adaptations to 12‐Week HIIT With or Without L‐Citrulline in Obese Older Adults

**DOI:** 10.1002/jcsm.70267

**Published:** 2026-04-03

**Authors:** Alexandre Mercier‐Guery, Marjorie Millet, Martine Croset, Vincent Marcangeli, Maude Dulac, Livia P. Carvalho, Guy Hajj‐Boutros, Pierrette Gaudreau, José A. Morais, Gilles Gouspillou, Philippe Noirez, Jean Charles Rousseau, Roland Chapurlat, Mylène Aubertin‐Leheudre

**Affiliations:** ^1^ INSERM UMR 1033, Université Claude Bernard‐Lyon 1, Hospices Civils de Lyon Lyon France; ^2^ Rheumatology Department Hôpital E. Herriot, Hospices Civils de Lyon Lyon France; ^3^ Département des sciences de l’activité physique Groupe de recherche en Activité Physique Adaptée, Faculté des Sciences, UQAM Montréal Québec Canada; ^4^ École de Réadaptation, Faculté de médecine et des sciences de la santé Université de Sherbrooke Sherbrooke Québec Canada; ^5^ Department of Medicine Research Institute of the McGill University Health Centre Montréal Québec Canada; ^6^ Département de Médecine de l’Université de Montréal, Centre de Recherche du Centre Hospitalier Universitaire de Montréal (CRCHUM) Université de Montréal Montréal Québec Canada; ^7^ Centre de Recherche de l’Institut Universitaire de Gériatrie de Montréal Montréal Québec Canada; ^8^ Meakins‐Christie Laboratories, Department of Medicine McGill University Montreal Quebec Canada; ^9^ PSMS, Université de Reims Champagne‐Ardenne Reims France

**Keywords:** adipose tissue, aerobic exercise, aging, biomarkers, dynapenia, gene expression, next‐generation sequencing, nutritional supplements, qPCR, sarcopenia

## Abstract

**Background:**

High‐intensity interval training (HIIT) with or without L‐citrulline (CIT) oral supplementation improves body composition, functional capacities and muscle health in obese older adults, potentially through microRNA‐driven regulation.

We aimed to (1) investigate the impact of a 12‐week HIIT on the expression level of microRNAs in vastus lateralis muscle biopsies and serum of obese older adults and (2) assess whether the differential expression level of microRNAs was associated with clinico‐biological adaptations to HIIT and provide potential biomarkers of HIIT response.

**Methods:**

In this secondary exploratory analysis of a double‐blind randomized trial, 36 women and 32 men (67.2 ± 5.2 years) following 12 weeks HIIT randomized in two groups were supplemented daily with CIT (HIIT‐CIT, *n* = 37) or with placebo (HIIT‐PLA, *n* = 31). Phenotypic variables, serum parameters, muscle biopsies and subcutaneous abdominal adipose tissue outcomes were collected pre‐intervention and postintervention. To assess the microRNA profile, the miRNome of muscle biopsy and serum was analysed using next generation sequencing in participants' subsets (*n* = 13). The microRNAs' differential expression level was analysed pre‐intervention and postintervention by TaqMan‐real‐time qPCR in 68 participants.

**Results:**

The expression of myo‐microRNAs (miR‐133a, b, ‐1, ‐206) and muscle‐related‐microRNAs (miR‐499, ‐208) was not altered following HIIT with or without CIT. In HIIT‐PLA, HIIT‐CIT and subgroups (based on sex, age, body mass index, dynapenic status), the change in muscle (miR‐504‐5p, ‐744‐5p, ‐151a‐3p, ‐106b‐5p, ‐127‐5p) and circulating (miR‐4433b‐5p, 151a‐3p, ‐744‐5p, 483‐3p, ‐106b‐5p, ‐484) microRNA levels was associated with changes of clinico‐biological parameters. Supplementing HIIT with CIT decreased the muscle miR‐504‐5p level (*p* = 0.022), correlating with lower body fat, improved functional capacities, muscle power and increased IGF‐1 level (*r* = −0.6, *p* < 0.05). MiR‐744‐5p expression increased in dynapenic participants (*p* = 0.04), associated with lean mass gain (*r* = 0.50, *p* < 0.05), while miR‐151a‐3p downregulation in men's muscle (*p* = 0.01) was associated with better insulin sensitivity (HOMA‐IR, *r* = 0.57, *p* = 0.05). In serum, miR‐151a‐3p upregulation in women (*p* = 0.01) correlated with improved muscle power and lower circulating leptin levels, while miR‐4433b‐5p downregulation (*p* = 0.001) was linked to reduced fat mass, lean mass gain and enhanced functional capacity. The downregulation of miR‐106b‐5p (*p* = 0.05) was associated with a higher adiponectin level and a better score of the 4‐m walking test (*p* = 0.05).

**Conclusion:**

HIIT did not impact the expression level of myo‐microRNAs and muscle‐related microRNAs but induced changes in muscle‐nonspecific microRNAs in muscle biopsy and serum. Modulations of microRNAs in muscle (miR‐504‐5p, ‐744‐5p) and serum (miR‐151a‐3p, ‐4433b‐5p, ‐106b‐5p) were associated with HIIT's beneficial effects, suggesting their role in the HIIT effects and their potential as candidate biomarkers for response.

## 1 Introduction

Aging, overweight/obesity, lack of physical activity and sedentary lifestyle are main factors negatively impacting muscle mass, strength and quality, increasing fat mass (FM) and intramuscular adipose tissue (AT) while impairing mitochondrial and glucose homeostasis [1, 2]. Aging and obesity induce metabolic disorders and alter signalling pathways and gene expression that overall lead to muscle wasting and impair muscle regeneration [2, 3]. Indeed, older adults with FM excess and loss of muscle strength (dynapenia) experience a decline in muscle function, functional and aerobic capacities [4, 5].

Physical exercise and nutrition are promising nonpharmacological interventions to counteract poor physical functions in obese older adults [4, 5, 6]. Different types of exercise training prevent the decline in muscle mass and muscle strength and result in clinical benefits for older adults [6, 7]. Specifically, high‐intensity interval training (HIIT), a subtype of endurance aerobic training, increases functional capacities, lean mass (LM), reduces waist circumference and improves the mitochondrial adaptations [8] A 12‐week HIIT reduces appendicular FM and increases LM in adult type 2 diabetic patients [S1]. In obese older adults, HIIT is slightly more effective than moderate‐intensity continuous training to improve functional capacities, maximal oxygen consumption, LM and skeletal markers of mitochondrial content, fusion and mitophagy [7]. Among nutritional strategies, the supplementation with the nonproteinogenic amino acid L‐citrulline (CIT), an intermediate of urea cycle produced from arginine, leads to body composition improvement in dynapenic or older adults and also in aged rats [9] [S2]. We have reported that adding CIT to HIIT results in a greater muscle strength increase and in a significant decrease in FM without altering the positive mitochondrial adaptations induced by HIIT in obese older adults [8].

The beneficial effects of various exercise types likely rely on multiple epigenetic regulations of signalling pathways that are altered in aging and in metabolic disorders, such as protein anabolism, mitochondrial homeostasis and adipocyte metabolism [2, 3, 10]. In this context, numerous studies have reported the effects of training programs on altered microRNA profiles in aging and in pathologies such as obesity and type II diabetes for which exercise is indicated as adjunctive therapy [11, 12]. These small regulatory RNAs, microRNAs, are posttranscriptional regulators of gene expression in muscle and AT that are stable in body fluids and easily detected in tissue and blood [13]. Exercise benefits might be associated with changes in blood microRNA profiles that reflect different organ contributions. In the context of aging and obesity, HIIT might trigger specific microRNA release from muscle and AT that might mediate its beneficial effects [14]. Due to their chemical stability and easy quantification in tissue and blood, microRNAs have been reported as biomarkers of the response to different training in various populations [15].

In this setting, we assessed the microRNA differential level of expression (differential LE) in *vastus lateralis* muscle biopsies and serum of groups of older men and women completing a 12‐week HIIT program combined with placebo (HIIT‐PLA) and supplemented with CIT (HIIT‐CIT) by analysing microRNAs LE after vs. before the intervention. These parameters were also analysed in all participants without supplement distinction (HIIT‐ALL) and in participant subsets according to sex, age, body mass index (BMI) and dynapenic status.

We also explored the underlying mechanisms of microRNAs' epigenetic regulations by correlating microRNAs' differential LEs with changes in physical performance, muscle function, body composition and blood parameters, following HIIT adaptations.

## 2 Material and Methods

### 2.1 Population and Study Design

This study is a secondary analysis including a subset of participants from a double‐blinded randomized interventional trial [7, 8] [S2]. To be included in this study, participants had to have extra serum samples available pre‐intervention and postintervention and to meet the previous inclusion criteria (see Supporting Information for more details). Of the included participants (36 women and 32 men, 67.2 ± 5.2 years), 45 were considered dynapenic (dynapenic criteria [handgrip strength]/body weight [BW]): women < 0.35 kg/kg; men < 0.42 kg/kg and 28 obese (based on BMI = 30 kg/m^2^) [8]. Among these participants, 28 of them had also an extra sample of *vastus lateralis* muscle biopsy.

To answer our objectives, we quantified microRNAs in muscle biopsy and serum of participant subsets at baseline and at the end of follow‐up in a discovery phase by next generation sequencing (NGS) (*n* = 13) and assessed the results of this initial phase in a larger validation phase in all participants by real‐time quantitative polymerase chain reaction (RT‐qPCR) (*n* = 68). Participants selected for the discovery phase did not differ from those included in the validation phase except for sex (Table S1).

**FIGURE 1:**
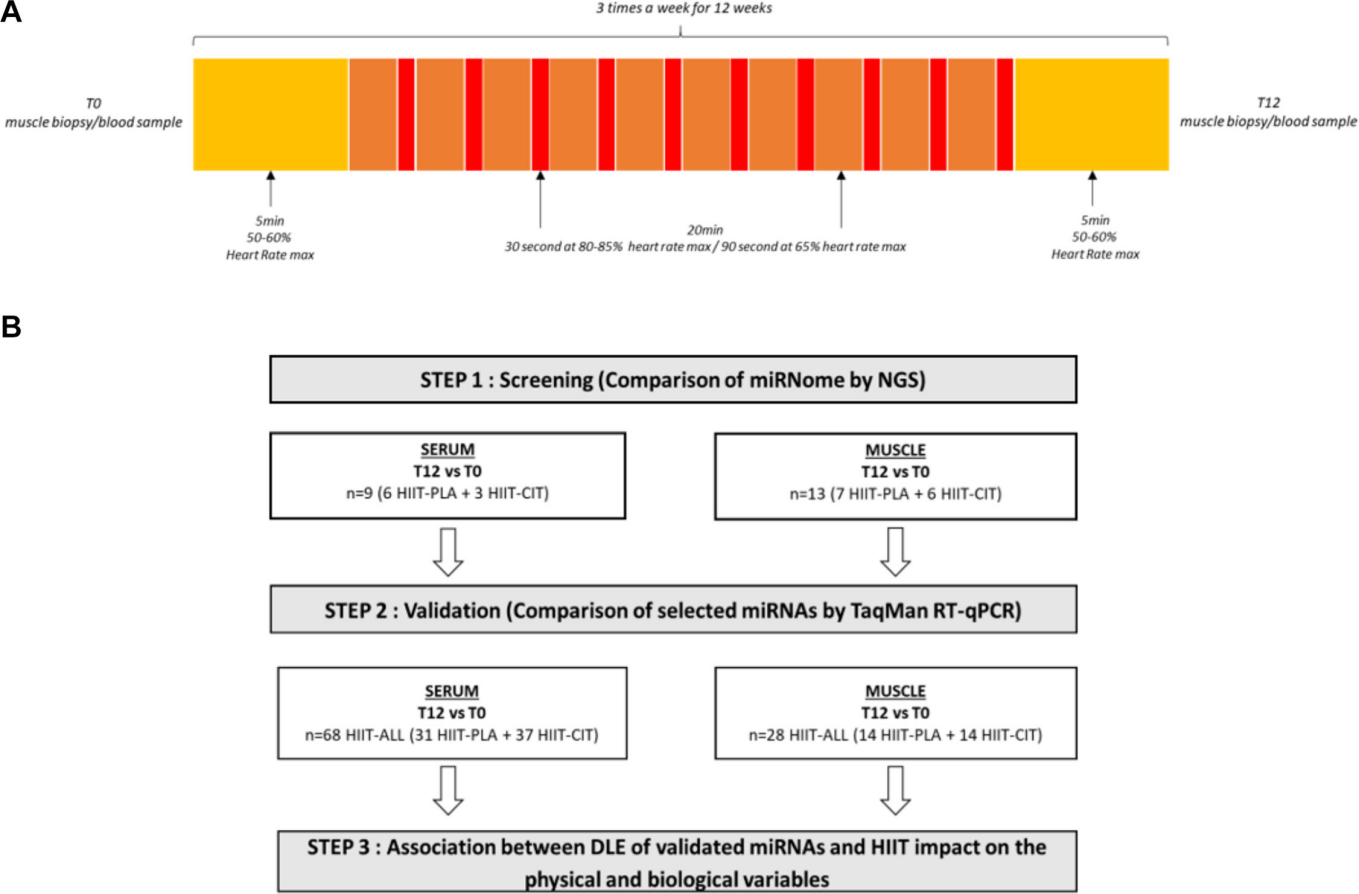
(1A) Organization scheme of the high‐intensity interval Training and the physical and biological parameters recording of participants. (1B) Flow‐chart of the study: muscle and serum microRNAs were quantified in HIIT‐PLA and HIIT‐CIT post‐ (T0) and pre‐ (T12) intervention by next generation sequencing and by TaqMan RT‐qPCR. Participants followed high‐intensity interval training (HITT) on an elliptical device three times per week on non‐consecutive days during 12 weeks. Participants in the HIIT+CIT group took a single daily dose of 10 g of L‐CIT (Citrage), while participants in the HIIT+PLA group took orally a single dose of a placebo powder (maltodextrin). The muscle strength, power, functional capacities and physical endurance, body composition, dietary intake and life habits were evaluated pre‐intervention and postintervention. At T0 and T12 (=after 12 weeks) participants had a blood sampling and vastus lateralis muscle biopsy. The measurements and samples ‘post HIIT’ were taken at least 48 h after the last session or at most 7 days after. DLE = differential level of expression.

As explained in detail previously [7, 8] [S2 and S3], all included participants completed a 12‐week HIIT on an elliptical device (3×/week for 12 weeks) in 30‐min supervised sessions: 5‐min warm‐up (≈ 50%–60% heart rate max [HRmax] or Borg's scale 8–12), 20 min of intervals consisting of repeated 30‐s sprints at ≈ 80%–85% HRmax or Borg > 17 interleaved with 90 s at ≈ 65% HRmax or Borg 13–16 and 5‐min cool‐down (50%–60% HRmax or Borg 8–12). Intensity was monitored with HR and perceived exertion, and speed/resistance were continuously adjusted to keep HR above target during the high‐intensity bouts; sessions adherence ≥ 80% was required. Exercise dose was conceptualized as the cumulative effect of 36 sessions (subject to adherence and free‐living activity). Muscle leg power and muscle leg power ratio were derived from the chair‐to‐stand test using the Takai sit‐to‐stand power index (Psit‐stand). Psit‐stand is computed from body mass, leg length, chair height (0.40 m) and the time to complete 10 sit‐to‐stand repetitions and is expressed in watts; the muscle leg power ratio corresponds to Psit‐stand normalized to body mass (W·kg^−1^) [16]. Participants were blindly randomly assigned to two groups: HIIT‐CIT (*n* = 37) took a daily dose of 10 g L‐CIT (Citrage) or HIIT‐PLA (*n* = 31) took a dose of a placebo powder (maltodextrin) equivalent in weight, appearance, taste and calories (38 kcal). Blood and tissue sampling were collected pre‐intervention and postintervention after an overnight fast. Participants had breakfast prior to functional capacities analyses recording (Figure 1A,B).

#### 2.1.1 Discovery Phase: miRNome Analysis by NGS

MicroRNA profiles were assessed by NGS in muscle biopsies (*n* = 13) and serum (*n* = 9). After total RNA extraction (miRNeasy Kits, QIAGEN) and library preparation (QIAseq microRNA Library Kit, QIAGEN), sequencing was performed on an Illumina NextSeq500 platform. For the sample quality controls of NGS analysis, see Supporting Information results. Reads were aligned to known microRNA sequences (miRBase21) and normalized using the TMM (Trimmed Mean of M‐values) method, with expression levels reported as Log_2_ fold changes (FCs) (Supporting Information methods) [S4].

#### 2.1.2 Validation Study: MicroRNAs by RT‐qPCR

Total RNA was extracted from muscle biopsies (*n* = 28) and serum samples (*n* = 68), with a synthetic spike‐in control (cel‐miR‐39‐3p) added. MicroRNAs were quantified using TaqMan Advanced microRNA technology on a QuantStudio 7 flex system (Applied Biosystems), and expression levels (Relative Quantification, RQ) were normalized according to the *ΔΔC*_*T*_ method and converted as FC = Log_2_(2^–ΔΔCT^) (Supporting Information methods) [S5 and S6].

#### 2.1.3 Subgroup Analyses

Pretraining vs. posttraining microRNA changes (T0 vs. T12) were compared for all participants (HIIT‐ALL). Further analyses explored the impact of citrulline (CIT) or placebo (PLA), as well as sex, age, dynapenic status and BMI on microRNA regulation (Supporting Information results).

### 2.2 Statistical Analysis

The clinical quantitative data were processed using the same methodology as in the previous studies (Supporting Information) [7, 8] [S2 and S3].

In the discovery phase, the miRNome was compared in the postintervention versus the pre‐intervention. In the validation phase, considering a risk for type‐II error due to limited sample size, we combined all participants in a single group (HIIT‐ALL) to examine the HIIT impact on microRNAs LEs [8]. The microRNAs LEs after training were compared to baseline in HIIT‐ALL, HIIT‐PLA, HIIT‐CIT and in the population subgroups. The NGS and differential LE analysis of microRNAs was performed by Qiagen using the EdgeR software package. Each differential LE was compared using an exact test analogous to Fisher's exact test but adapted for overdispersal data. The *p*‐values were corrected for false discovery rate by the Benjamini–Hochberg method and were considered for values *q* < 0.05.

For RT‐qPCR, the Wilcoxon matched pairs signed rank test was used to examine the significance of differences in muscle and serum microRNAs levels in participants (T12 vs. T0). A *p*‐value < 0.05 was considered significant.

Spearman's correlation coefficient was performed to assess the association between microRNAs' differential LEs and clinico‐biological parameters changes. The *p*‐values from correlation analyses were adjusted for multiple testing using Holm's step‐down procedure.

The analyses, graphics and figures were performed using GraphPad Prism version 8.0.0 for Windows, GraphPad Software, San Diego, California USA (http://www.graphpad.com).

## 3 Results

### 3.1 Effects of HIIT and Supplementation on Population Characteristics

The main characteristics of the population at baseline did not differ significantly between groups and the interventions did not change BW or BMI within or between groups (Table 1). Total and leg LM as well as physical performance were significantly improved in HIIT‐PLA and HIIT‐CIT. No significant changes in energy balance were reported except for a significant decrease in fat intake in HIIT‐PLA (Table 1). The clinical and biological parameters of our population were similar to the previous main study [8].

**TABLE 1:** The differences in body composition, physical performances, muscle function and composition, blood parameters and in energy balance before and after 12‐week HIIT in participants supplemented with a placebo (HIIT‐PLA) and in participants supplemented with L‐citrulline (HIIT‐CIT).

Variables	HIIT‐PLA (*n* = 31)			HIIT‐CIT (*n* = 37)			Time	Group	Time*group
	Pre	Post	*p*	Pre	Post	*p*			
Body composition (DXA)									
Body weight (kg)	82.2 ± 13.5	82.2 ± 13.4	0.92	79.5 ± 13.5	78.8 ± 13.3	0.07	0.20	0.35	0.19
BMI (kg/m^2^)	30.0 ± 5.1	30.0 ± 5.0	0.98	29.3 ± 4.6	29.2 ± 4.8	0.61	0.70	0.52	0.74
Total LBM (kg)	47.9 ± 9.3	48.6 ± 9.3	0.02	46.3 ± 8.0	46.9 ± 8.3	0.02	< 0.0001	0.43	0.81
Leg LM (kg)	17.1 ± 3.5	17.4 ± 3.7	0.02	16.3 ± 2.8	16.6 ± 2.8	0.02	0.001	0.34	0.84
Appendicular LM (kg)	22.7 ± 5.2	22.9 ± 5.4	0.13	21.6 ± 4.2	21.8 ± 4.2	0.07	0.02	0.33	0.91
Total MMI (kg/m^2^)	17.4 ± 2.4	17.6 ± 2.7	0.07	17.0 ± 2.0	17.3 ± 2.4	0.008	0.002	0.54	0.64
Appendicular MMI (kg/m^2^)	8.2 ± 1.3	8.3 ± 1.4	0.29	7.9 ± 1.1	8.0 ± 1.1	0.02	0.02	0.77	0.73
Total FM (%)	37.6 ± 7.8	37.2 ± 7.8	0.24	37.9 ± 6.6	36.7 ± 6.6	0.0005	0.0009	0.94	0.12
Android FM (%)	47.1 ± 7.3	46.7 ± 7.3	0.45	46.9 ± 8.0	45.6 ± 8.1	0.006	0.01	0.73	0.18
Gynoid FM (%)	39.7 ± 10.3	39.7 ± 10.4	0.94	40.6 ± 9.0	39.5 ± 8.9	0.02	0.09	0.86	0.14
Leg FM (%)	35.6 ± 11.0	35 ± 11.1	0.21	36.4 ± 9.5	35.2 ± 9.3	< 0.0001	0.001	0.86	0.17
Physical performances									
Normal 4‐m walking speed (m/s)	2.99 ± 0.38	2.72 ± 0.3	< 0.0001	2.97 ± 0.38	2.73 ± 0.31	< 0.0001	< 0.0001	0.97	0.8
Rapid 4‐m walking speed (m/s)	2.08 ± 0.25	1.94 ± 0.24	0.0005	2.12 ± 0.37	1.91 ± 0.28	< 0.0001	< 0.0001	0.92	0.2
6‐min walking test (m)	555 ± 82	617 ± 90	< 0.0001	546 ± 94	620 ± 96	< 0.0001	< 0.0001	0.8	0.61
Estimated V02 max (mL/min/kg)	17.7 ± 1.9	19.1 ± 2.1	< 0.0001	17.6 ± 2.1	19.2 ± 2.2	< 0.0001	< 0.0001	0.91	0.65
5‐rep chair test (s)	19.8 ± 5.1	16.0 ± 4.0	< 0.0001	19.1 ± 3.4	15.5 ± 2.9	< 0.0001	< 0.0001	0.60	0.61
Alternate step test (*n*)	28.8 ± 4.1	33.1 ± 5.0	< 0.0001	29.7 ± 5.2	33.5 ± 5.5	< 0.0001	< 0.0001	0.62	0.39
Normal 3‐m TUG (s)	10.21 ± 1.4	9.13 ± 1.19	< 0.0001	10.1 ± 1.63	8.74 ± 1.12	< 0.0001	< 0.0001	0.39	0.42
Rapid 3mTUG (s)	7.51 ± 1.07	6.71 ± 0.92	< 0.0001	7.41 ± 1.09	6.37 ± 1.0	< 0.0001	< 0.0001	0.39	0.22
Muscle function									
HS (kg)	33.2 ± 10.6	34.3 ± 10.4	0.65	32.3 ± 9.4	34.9 ± 10.9	0.25	0.26	0.94	0.66
HSr (kg/BWkg)	0.40 ± 0.12	0.42 ± 0.1	0.63	0.4 ± 0.1	0.44 ± 0.11	0.10	0.13	0.57	0.44
LLMS (kg)	345 ± 103	354 ± 103	0.50	327 ± 94	370 ± 99	< 0.0001	0.0006	0.94	0.03
LLMSr (kg/BWkg)	4.20 ± 1.14	4.34 ± 1.02	0.56	4.12 ± 0.98	4.68 ± 0.98	< 0.0001	0.0003	0.53	0.01
Muscle leg power (W)	160 ± 74	188 ± 74	< 0.0001	154 ± 60	183 ± 66	< 0.0001	< 0.0001	0.68	0.94
Muscle leg power ratio (W.BWkg)	1.94 ± 0.73	2.27 ± 0.72	0.0002	1.93 ± 0.67	2.25 ± 0.79	< 0.001	< 0.0001	0.94	0.01
Muscle composition (QPCT)									
Subcutaneous FM area	83.0 ± 49.5	80.8 ± 46.2	0.23	75.2 ± 39.2	70.9 ± 35.1	0.05	0.03	0.48	0.62
Intramuscular FM area	5.0 ± 2.2	4.8 ± 2.3	0.77	4.7 ± 2.8	4.0 ± 2.2	0.12	0.19	0.35	0.39
Blood parameters									
Insulin (pmol/L)	48.3 ± 23.3	48.4 ± 27.6	0.99	51.3 ± 27.8	51.9 ± 27.3	0.96	0.97	0.62	0.98
Glucose (mmol/L)	6.00 ± 1.30	6.08 ± 1.50	0.29	5.53 ± 0.73	5.68 ± 0.64	0.05	0.04	0.10	0.55
HOMA IR	2.16 ± 1.16	2.19 ± 1.36	0.84	2.15 ± 1.28	2.30 ± 1.45	0.49	0.53	0.92	0.75
QUICKI	0.351 ± 0.035	0.352 ± 0.036	0.86	0.352 ± 0.035	0.349 ± 0.033	0.42	0.65	0.94	0.50
NEFA (mmol/L)	0.50 ± 0.16	0.45 ± 0.17	0.14	0.45 ± 0.18	0.44 ± 0.15	0.73	0.21	0.41	0.39
Adiponectin (μg/mL^)^	14.1 ± 7.7	13.9 ± 7.8	0.79	14.1 ± 8.7	14.5 ± 10.4	0.55	0.79	0.87	0.56
IGF‐1 (ng/mL^)^	86.2 ± 19.3	91.3 ± 21.0	0.08	91.4 ± 28.8	94.1 ± 32.4	0.32	0.06	0.51	0.55
IGFBP‐3 (ug/mL^)^	1.84 ± 0.36	1.94 ± 0.33	0.02	1.94 ± 0.47	1.92 ± 0.48	0.51	0.24	0.70	0.03
Dietary intakes – Physical activity level									
Total energy intake (kcal/day)	2063 ± 718	1865 ± 509	0.14	1922 ± 541	1823 ± 533	0.40	0.12	0.40	0.60
Protein intake (g/day)	84.4 ± 20.1	82.8 ± 19.8	0.78	82.9 ± 28.7	83.5 ± 28.5	0.93	0.90	0.93	0.80
Carbohydrate intake (g/day)	246 ± 85	227 ± 67	0.22	229 ± 70	213 ± 64	0.22	0.10	0.29	0.92
Lipid intake (g/day)	83.5 ± 46.6	66.0 ± 25.1	0.03	75.3 ± 31.2	69.8 ± 28.3	0.43	0.04	0.71	0.27
Number of steps (*n*/day)	6441 ± 3163	5812 ± 3660	0.18	6182 ± 3235	5937 ± 2938	0.85	0.43	0.94	0.28

*Note:* Data are means ± SD. The *p*‐value: significant intragroup differences between pre‐intervention and postintervention using paired *t*‐test. Results were considered statistically significant when *p*‐value < 0.05. Time (HIIT intervention) and time × group effects using repeated‐measure ANOVA. Levene's test was used to assess the homogeneity of variances. A linear mixed‐models approach with a two‐factor repeated measures ANOVA was then used to test the intervention effect (time effect: T0 and T12), the supplementation effect (supp effect: PLA and CIT) and their interaction (Time×Supp effect) on the outcomes. BW: body weight; BMI: body mass index; FM: fat mass; LM: lean mass; LBM: lean body mass; MMI: muscle mass index; TUG: Timed Up and Go; HS: handgrip strength; HSr: relative handgrip strength (HS/body weight); LLMS: lower limb muscle strength; LLMSr: relative lower limb muscle strength (LLMS/body weight). Homeostasis model of assessment of insulin resistance (HOMA‐IR) index was calculated using the following equation: [glycemia mmol/L × insulinemia μU/mL]/22.5. QUICKI index was calculated using the following equation: 1/([log glycemia mg/dL + log insulinemia μU/mL]); NEFA: nonesterified fatty acids; IGF‐1: Insulin‐like growth factor1, IGFBP‐3: insulin‐like growth factor‐binding protein‐3. Metabolic syndrome was defined as having at least three components including waist circumference (WC) criteria; sarcopenic status was defined using the following validated equation: appendicular (leg + arms) lean mass/height (m^2^) and these criteria for women: < 5.5 kg/m^2^ and for men: < 7.7 kg/m^2^; dynapenic status was defined using the following validated equation: HS/BW (kg/kg) and these criteria for women: < 0.35 kg/kg and for men: < 0.42 kg/kg. BMI obesity criteria were defined as having a BMI>or equal to 30 kg/m2; WC/HC (waist/hip circumference) ratio metabolic criteria were defined as having a ratio>or equal to 1.

### 3.2 Discovery Phase: Serum and Muscle microRNA Profiling

We conducted this phase by NGS that enables the analysis of global microRNAs expression and sequence to quantify and compare their LE in muscle biopsy and serum within the different groups of participants. The NGS analysis identified 210 microRNAs expressed with level > 10 TMM in muscle biopsies from the subsample (*n* = 13 participants). Based on NGS quantification, the myo‐microRNAs (miR‐133a‐3p, ‐133b, ‐1, ‐206) represented around 60% of microRNAs detected in muscle biopsies (Table S3). This high myo‐microRNAs expression was restricted to muscle tissue since only low levels were detected in serum. The members of muscle‐related microRNAs (miR‐208a, ‐208b, ‐499) representing less than 1% of total muscle microRNAs were detected as traces in serum.

#### 3.2.1 Muscle Biopsy NGS Analysis

**FIGURE 2:**
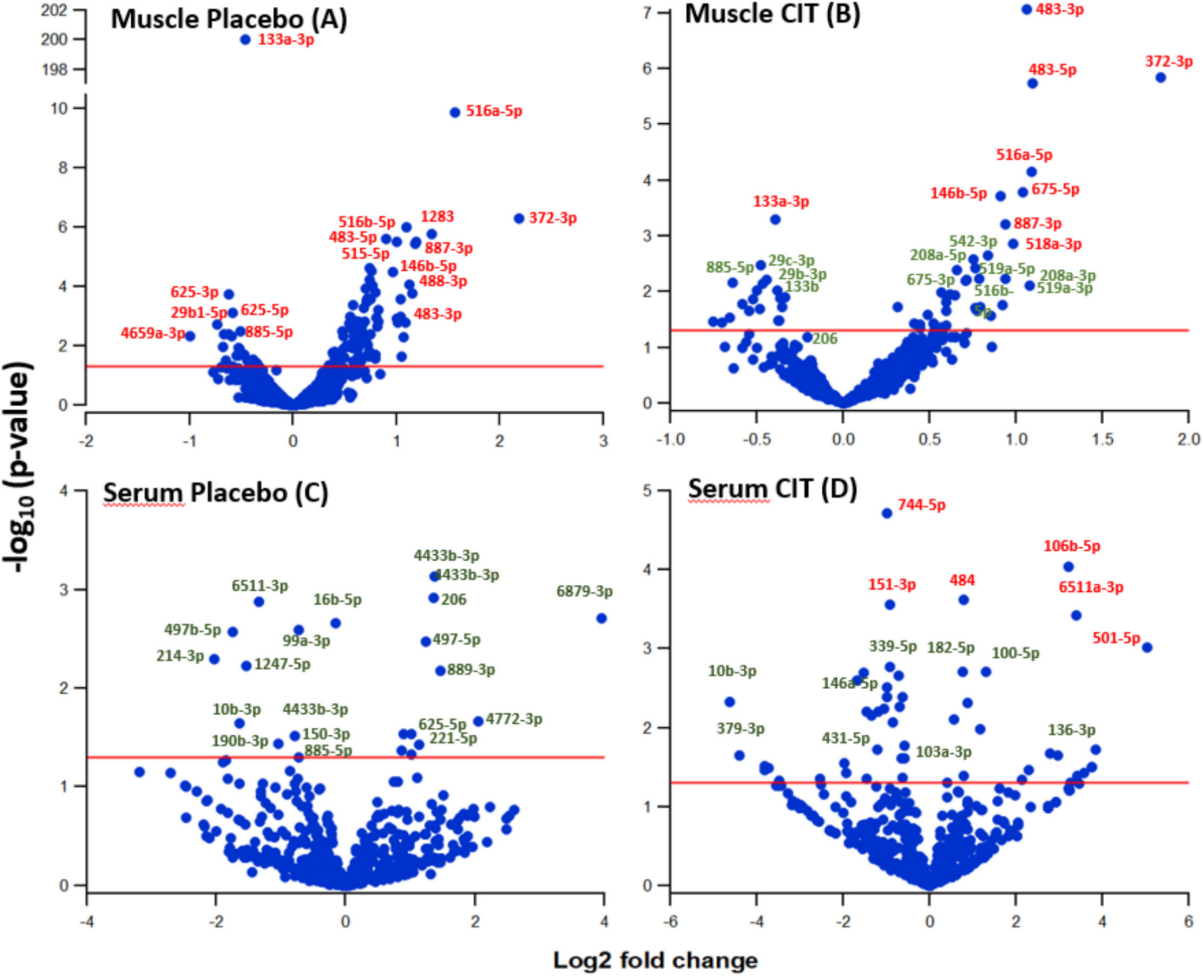
Volcano plots representing the relationship between the *p*‐values and the fold change of the microRNAs normalized expression in muscle and serum of dynapenic‐obese older adults under High‐Intensity Interval Training supplemented with placebo (2A, 2C) or with L‐citrulline (2B, 2D). The graphs are constructed by plotting the *p*‐values (−log_10_ *p*‐values) on the *y*‐axis and the expression fold change (Log_2_ FC) before and after the 12‐weeks HIIT on the *x*‐axis. The microRNAs indicated in green have FDR corrected *p* < 0.05. The microRNAs that are found towards the top of the plot are high significant values and the microRNAs at the extreme left or right are downregulated or upregulated, respectively. The microRNAs in red were significantly different after FDR correction.

The microRNA profiling was compared postintervention vs. pre‐intervention, by plotting the microRNAs differential LE (Log_2_FC) vs. the statistical significance of changes (−log_10_(p‐values)) in HIIT‐PLA (*n* = 7) and in HIIT‐CIT (*n* = 6). Volcano plots showed that 59 microRNAs were differentially expressed after FDR‐corrected *p*‐values in HIIT‐PLA. Among these, 9/59 microRNAs were downregulated, and 16/59 microRNAs were upregulated with a Log_2_FC > 1 (Figure 2A, Table S4‐1A). In HIIT‐CIT, within the 60 microRNAs that were significantly differently expressed after the intervention (*p* < 0.05), 17/60 were downregulated and 8/60 upregulated with Log_2_FC > 1 (Figure 2B and Table S4‐1B). After FDR correction, the LE of 7 and 8 microRNAs remained significantly modulated after exercise in HIIT‐PLA and HIIT‐CIT, respectively. Five microRNAs differed significantly after 12‐week HIIT in muscle biopsies of both groups with miR‐133a‐3p being downregulated and miR‐516a, miR‐372‐3p, miR‐483‐5p and miR‐887‐3p being upregulated (Table 2).

**TABLE 2:** The screening phase analysed by next generation sequencing for comparison of microRNAs differential expression in participants supplemented with placebo (muscle biopsy = 7, serum = 6) or supplemented with L‐citrulline (muscle biopsy = 6, serum = 3) before and after 12‐week HIIT.

	MicroRNAs	T0 (TMM)	T12 (TMM)	Log2 FC	*p*	*Q*‐value
MUSCLE HIIT PLA	hsa‐miR‐133a‐3p	251400.84	182131.02	−0.458898	< 1 × 10^−10^	< 1 × 10^−10^
	hsa‐miR‐516a‐5p	8.06	21.96	1.56865	1.3 × 10^−10^	3.8 × 10^−8^
	hsa‐miR‐372‐3p	0.46	1.81	2.18059	4.9 × 10^−7^	9.3 × 10^−5^
	hsa‐miR‐516b‐5p	4.82	9.15	1.09771	1 × 10^−6^	1.4 × 10^−4^
	hsa‐miR‐1283	1.4	3.45	1.33744	1.7 × 10^−6^	2 × 10^−4^
	hsa‐miR‐483‐5p	16.68	30.21	0.897008	2.4 × 10^−6^	2.1 × 10^−4^
	hsa‐miR‐887‐3p	2.12	4.65	1.19403	3.0 × 10^−6^	2.1 × 10^−4^
	hsa‐miR‐515‐5p	6.3	12.22	1.00503	3.0 × 10^−6^	2.1 × 10^−4^
	hsa‐miR‐488‐3p	1.1	2.56	1.17635	3.7 × 10^−6^	2.3 × 10^−4^
MUSCLE HIIT CIT	hsa‐miR‐483‐5p	14.14	29.08	1.062	8.6 × 10^−8^	4.9 × 10^−5^
	hsa‐miR‐372‐3p	0.46	1.6	1.836	1.4 × 10^−6^	3.4 × 10^−4^
	hsa‐miR‐483‐3p	53.06	107.85	1.094	1.8 × 10^−6^	3.4 × 10^−4^
	hsa‐miR‐516a‐5p	13.35	23.24	1.088	7.0 × 10^−5^	0.010
	hsa‐miR‐675‐5p	2.23	4.39	1.039	1.6 × 10^−4^	0.018
	hsa‐miR‐146b‐5p	54.35	101.88	0.909	1.9 × 10^−4^	0.018
	hsa‐miR‐133a‐3p	247553.79	186742.73	−0.393	5.1 × 10^−4^	0.042
	hsa‐miR‐887‐3p	1.98	3.83	0.940	6.2 × 10^−4^	0.044
	hsa‐miR‐518a‐3p	1.18	2.43	0.982	0.0014	0.086
SERUM HIIT PLA	hsa‐miR‐4433b‐3p	13.22	36.96	1.37995	7.4 × 10^−4^	0.265
	hsa‐miR‐206	45.25	167.24	1.35772	0.0012	0.265
	hsa‐miR‐6511b‐3p	27.39	10.61	−1.34634	0.0013	0.265
	hsa‐miR‐6879‐3p	0	5.91	3.95485	0.0020	0.265
	hsa‐miR‐16‐5p	205984.2	190 494	−0.152022	0.0022	0.265
	hsa‐miR‐99a‐5p	255.96	143.1	−0.723232	0.0026	0.265
	hsa‐miR‐497‐5p	16.47	4.67	−1.75577	0.0027	0.265
	hsa‐miR‐5187‐5p	11.08	28.59	1.2413	0.0033	0.285
	hsa‐miR‐214‐3p	11.48	2.47	−2.03014	0.0051	0.387
SERUM HIIT CIT	hsa‐miR‐744‐5p	1896.46	961.74	−0.999376	1.9 × 10^−5^	0.011
	hsa‐miR‐106b‐5p	5.53	64.18	3.20741	9.2 × 10^−5^	0.026
	hsa‐miR‐484	925.9	1609.82	0.782608	2.4 × 10^−4^	0.038
	hsa‐miR‐151a‐3p	3351.23	1846.92	−0.913021	2.7 × 10^−4^	0.038
	hsa‐miR‐6511a‐3p	2.77	36.83	3.38396	3.8 × 10^−4^	0.042
	hsa‐miR‐501‐5p	0	21.28	5.04169	9.5 × 10^−4^	0.090
	hsa‐miR‐339‐5p	1258.67	721.8	−0.920887	0.0017	0.111
	hsa‐miR‐100‐5p	45.74	120.23	1.31214	0.0019	0.111
	hsa‐miR‐182‐5p	799.27	1374.79	0.758977	0.0019	0.111

*Note:* The microRNAs identification and quantification were performed by NGS analysis. Absolute microRNAs amount was expressed as the Tags per Million mapped reads normalized by the trimmed mean of M‐values (TMM). The fold change (Log2 FC) of the most differentially expressed microRNAs was reported in muscle and serum before (T0) and after 12‐week HIIT (T12) in HIIT‐PLA (PLA) and HIIT‐CIT (CIT) groups. The differential expression analysis was performed using the EdgeR statistical software package (Bioconductor) with a TMM normalization based on the log‐fold and absolute values changes in expression levels between samples. The *p*‐values were estimated by an exact test on the negative binomial distribution and corrected by the Benjamini‐Hochberg FDR (q‐values).

#### 3.2.2 Serum NGS Analysis

The miRNome analysis conducted in serum (*n* = 9 participants) identified 21 and 52 microRNAs that were differentially expressed in HIIT‐PLA and HIIT‐CIT (*p* < 0.05), respectively. After FDR correction, none of them (0/21) differed significantly in HIIT‐PLA and 5 (5/52) in HIIT‐CIT remained significantly different between pre‐intervention and postintervention (Figure 2C,D and Table S4C,D).

### 3.3 Validation Phase: Differential Expression of Candidate microRNAs in Muscle and Serum

Nineteen microRNAs were selected for further RT‐qPCR analysis based on their significant differential expression between groups, high NGS read level (Table S5) and on their potential regulation of signalling pathways in response to HIIT, according to their experimentally validated interactions with targeted mRNAs (Table S6).

MicroRNA LE has been compared (T12 vs. T0) in HIIT‐ALL, HIIT‐PLA and HIIT‐CIT and in population subgroups divided according to sex, age, BMI or dynapenic status.

#### 3.3.1 Muscle Biopsies

The myo‐microRNA, miR‐133a‐3p tended to be downregulated in HIIT‐CIT, whereas it tended to increase in HIIT‐PLA and HIIT‐ALL (Table 3). Its LE did not significantly differ in population subgroups (Table S7).

**TABLE 3:** The comparison of microRNA levels by RT‐qPCR in muscle biopsy and serum of participants supplemented with either placebo or L‐citrulline after and before 12‐week HIIT.

		HIIT‐ALL			HIIT‐PLA			HIIT‐CIT		
	microRNA	T0	T12	*p*	T0	T12	*p*	T0	T12	*p*
MUSCLE BIOPSY	hsa‐483‐5p	−0.355	0.235	0.142	−0.036	0.235	0.380	−0.483	0.198	0.320
	hsa‐483‐3p	−0.342	0.250	0.290	−0.158	0.085	0.735	−0.535	0.361	0.268
	hsa‐516a‐5p	−0.445	0.579	0.218	−0.141	0.303	0.970	−0.938	0.593	0.129
	hsa‐369‐3p	0.277	−0.304	0.134	0.501	−0.465	0.380	0.067	0.070	0.278
	hsa‐136‐3p	0.058	−0.165	0.695	0.406	−0.036	0.715	−0.377	−0.276	0.903
	hsa‐146b‐5p	−0.569	−0.778	0.922	−0.569	−1.567	0.625	0.343	2.448	0.688
	hsa‐133a‐3p	0.088	0.208	0.537	0.088	0.512	0.296	0.069	−0.367	0.952
	hsa‐504‐5p	0.232	−0.005	0.041*	0.066	0.106	0.999	0.708	−0.646	0.022*
	hsa‐1277‐5p	0.561	−0.948	0.414	−0.570	−1.305	0.652	0.822	−0.067	0.375
	hsa‐136‐5p	0.255	0.281	0.715	−0.168	0.673	0.296	0.255	0.281	0.715
	hsa‐181a‐3p	0.274	0.335	0.325	0.312	0.776	0.424	0.269	0.290	0.542
	hsa‐625‐3p	0.537	−0.381	0.207	0.537	−0.003	0.432	0.859	−0.682	0.297
	hsa‐515‐5p	0.217	0.044	0.924	−0.323	−0.369	0.465	0.503	0.598	0.413
	hsa‐127‐5p	0.230	0.597	0.374	0.626	0.670	0.831	−0.577	0.524	0.275
	hsa‐744‐5p	0.198	0.154	0.327	0.535	0.264	0.903	−0.042	0.099	0.241
	hsa‐484	−0.095	−0.174	0.552	−0.051	−0.143	0.135	−0.230	−0.206	0.670
	hsa‐151a‐3p	0.374	−0.226	0.062	0.111	0.052	0.414	0.478	−0.356	0.079
	hsa‐4433b‐5p	NA	NA	NA	NA	NA	NA	NA	NA	NA
	hsa‐106b‐5p	0.272	0.241	0.582	0.620	0.325	0.153	−0.558	−0.174	0.358
SERUM	hsa‐483‐5p	0.327	0.536	0.773	0.055	0.603	0.949	0.437	0.333	0.653
	hsa‐483‐3p	−0.406	−0.482	0.044*	−0.147	−0.685	0.244	−0.813	−0.261	0.094
	hsa‐516a‐5p	NA	NA	NA	NA	NA	NA	NA	NA	NA
	hsa‐369‐3p	0.257	−0.067	0.239	−0.039	0.431	0.747	0.523	−0.130	0.074
	hsa‐136‐3p	−0.370	0.374	0.737	0.334	0.978	0.243	−0.509	−0.417	0.439
	hsa‐146b‐5p	NA	NA	NA	NA	NA	NA	NA	NA	NA
	hsa‐133a‐3p	0.499	−0.091	0.858	0.881	−0.278	0.541	0.193	0.012	0.246
	hsa‐504‐5p	NA	NA	NA	NA	NA	NA	NA	NA	NA
	hsa‐1277‐5p	NA	NA	NA	NA	NA	NA	NA	NA	NA
	hsa‐136‐5p	0.457	−0.335	0.410	0.334	−0.176	0.850	0.415	−0.335	0.365
	hsa‐181a‐3p	0.401	0.483	0.646	0.527	0.623	0.870	0.356	0.483	0.368
	hsa‐625‐3p	−0.093	−0.139	0.164	0.373	−0.896	0.329	−0.184	0.136	0.320
	hsa‐515‐5p	NA	NA	NA	NA	NA	NA	NA	NA	NA
	hsa‐127‐5p	NA	NA	NA	NA	NA	NA	NA	NA	NA
	hsa‐744‐5p	0.703	0.455	0.004*	0.537	0.285	0.006*	0.848	0.455	0.189
	hsa‐484	0.440	−0.215	0.078	0.495	−0.435	0.025*	0.217	−0.073	0.846
	hsa‐151a‐3p	−0.347	0.455	0.001*	−0.337	0.612	0.019*	−0.358	0.313	0.014*
	hsa‐4433b‐5p	0.860	−0.759	0.001*	0.860	−0.006	0.060	0.905	−1.312	0.004*
	hsa‐106b‐5p	0.847	−0.337	0.1077	1.031	−0.359	0.039*	0.749	−0.319	0.882

*Note:* The 19 candidate microRNAs were analysed by TaqMan RT‐qPCR in muscle biopsy and in serum of HIIT‐ALL (muscle biopsies *n* = 28; serum *n* = 68), HIIT‐PLA (muscle biopsy *n* = 14, serum *n* = 37) and HIIT‐CIT (muscle biopsy *n* = 14, serum *n* = 31). After normalization, the microRNAs levels were expressed as relative values calculated as 2^–ΔΔCT^, with ΔCT = (CT microRNA – CT mean of endogenous controls) and ΔΔCT = (ΔCT of the microRNA–ΔCT mean of microRNA through all samples) and converted as Fold Change (FC) = Log2(2–^ΔΔCT^). A comparison of medians was performed between groups by the Wilcoxon test. Results were considered statistically significant when *p*‐value < 0.05 (*).

MiR‐504‐5p was downregulated in muscle biopsy of HIIT‐CIT (*p* = 0.022) and in HIIT‐All (*p* = 0.041) with a differential LE higher in HIIT‐CIT than in HIIT‐PLA (−1.31 vs. −0.07, *p* = 0.02) (Table 3, S7). Its LE (T12) was lower in HIIT‐CIT than in HIIT‐PLA (−0.65 vs. 0.11, *p* = 0.022, Table S7).

The miR‐146b‐5p upregulation in HIIT‐CIT that was significant in the screening phase did not reach significance in this validation phase. However, its LE was higher in HIIT‐CIT vs. HIIT‐PLA at the end of the intervention (2.45 vs. −1.57, *p* = 0.036) (Table S7).

**FIGURE 3:**
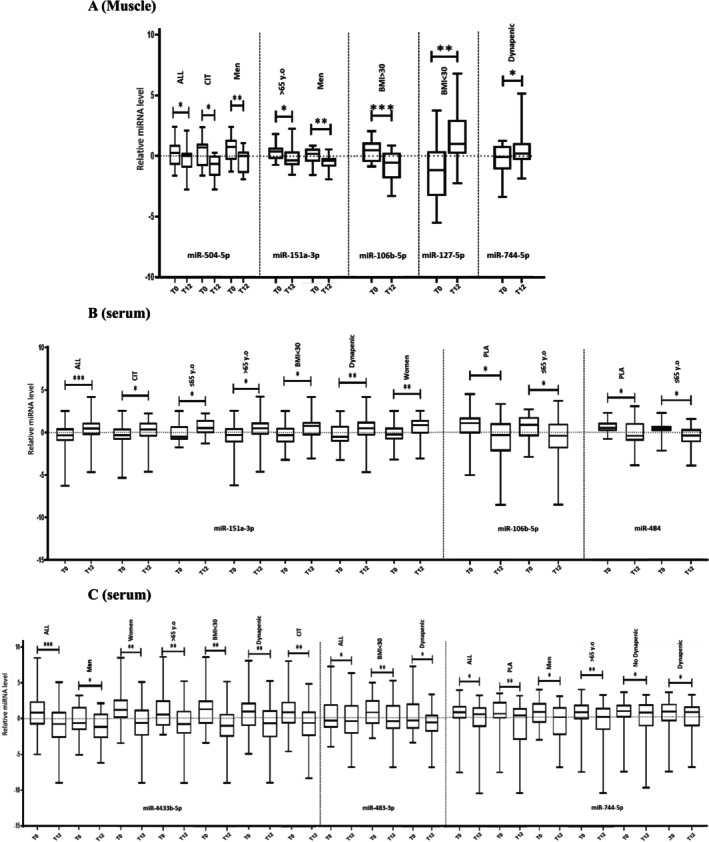
The relative levels of the 19 microRNAs that were significantly different after 12‐week HIIT in dynapenic‐obese older adults supplemented with placebo or with L‐citrulline in muscle biopsy (3A) and in serum (3B, 3C). From the screening phase, 19 microRNAs were selected for validation and further analysed by RT‐qPCR. Their relative level expressed as median was compared between pre‐ (T0) and post‐ (T12) intervention, by the Wilcoxon test. (*p‐value ≤ 0.05; **p‐value ≤ 0.01; ***p‐value ≤ 0.001). ALL, PLA, CIT, men, women, > / < 65 years (y.o), BMI < / > 30 and dynapenic represent the subset of participants, according to sex, age, body mass index, supplemented with L‐citrulline (CIT) or with placebo (PLA) and in all participants completing the 12‐week HIIT (ALL).

Regarding the other subgroups analysis, we also found that miR‐151a‐3p LE decreased after the intervention in participants aged > 65 years old (*p* = 0.04) and in men (*p* = 0.01) but was not altered by the BMI or dynapenic status (Figure 3A). This response was stimulated by the CIT supplementation in men only (*p* = 0.011). We observed a significant decrease for miR‐106b‐5p LE in participants with a BMI > 30 kg/m^2^ (*p* = 0.001), an upregulation of miR‐127‐5p in those with a BMI < 30 kg/m^2^ (*p* = 0.009) and of miR‐744‐5p in dynapenic participants (*p* = 0.04) (Figure 3A).

#### 3.3.2 Serum

The circulating myo‐microRNA miR‐133a‐3p was weakly expressed and was downregulated in HIIT‐PLA and HIIT‐CIT, although not significantly (Table 3). Moreover, its differential LE did not significantly differ in population subgroups (Table S7).

The muscle‐nonspecific microRNAs LE (miR‐4433b‐5p, ‐151a‐3p, ‐483‐3p, ‐106b‐5p, ‐744‐5p, ‐106b‐5p, ‐484) was significantly modulated after intervention. The miR‐151a‐3p LE was increased in HIIT‐ALL (*p* = 0.001), in HIIT‐PLA (*p* = 0.019) and in HIIT‐CIT (*p* = 0.014). In HIIT‐ALL, miR‐483‐3p and miR‐744‐5p were downregulated (*p* = 0.044, *p* = 0.004, respectively). The miR‐4433b‐5p was downregulated in HIIT‐ALL (*p* = 0.001) and HIIT‐CIT (*p* = 0.004). In contrast, miR‐106b‐5p, miR‐484 and miR‐744‐5p were significantly downregulated in HIIT‐PLA (Table 3). The LE of all microRNAs did not significantly differ between HIIT‐PLA and HIIT‐CIT at T12, except for miR‐136‐3p which was lower postintervention in HIIT‐CIT vs. HIIT‐PLA (−0.16 vs. 0.98, *p* = 0.016, Table S7).

Regarding subgroup analysis, the miR‐151a‐3p LE was increased irrespective of age (*p* = 0.003 and *p* = 0.022 in < 65 years‐old and > 65 years‐old, respectively), in men (*p* = 0.003), in participants with a BMI < 30 kg/m^2^ (p = 0.003) and with dynapenia (*p* = 0.005). The miR‐483‐3p, which did not significantly differ in HIIT‐PLA and HIIT‐CIT, was decreased in participants with a BMI < 30 kg/m^2^ (−0.48 vs. 0.75, *p* = 0.009) and with dynapenia (−0.69 vs. −0.35, *p* = 0.018) (Figure 3B). The miR‐4433‐5p was significantly downregulated in women, men, in participants aged > 65 years‐old, dynapenic or with a BMI < 30 kg/m^2^ (*p* < 0.05) (Figure 3C). MiR‐744‐5p was significantly downregulated in men, in participants aged > 65 years‐old, with a BMI > 30 kg/m^2^ and with or without dynapenia (*p* < 0.05) (Figure 3C, Table S7). Finally, miR‐106b‐5p and miR‐484 were significantly downregulated in HIIT‐PLA & in participants aged < 65 years‐old (Figure 3B).

### 3.4 Validated Target Genes of Differentially Expressed microRNAs

To decipher the microRNA‐mediated regulations of signalling pathways involved in response to intervention, we searched for potential interactions of these microRNAs with targeted mRNAs using the Web‐based computational databases to identify the microRNA interactions with their predicted and previously reported experimentally validated target genes (Table S6).

Among the microRNAs analysed in the validation phase, the miR‐133a‐3p and 3 muscle‐unspecific microRNAs (miR‐483‐3p, 106b‐5p, ‐4433b‐5p) target IGF‐1/IGF‐1‐R and may impact IGF‐1/IGF‐1‐R/‐R2 and AKT/mTOR/S6k signalling involved in protein anabolism (mTORC1), breakdown (FOXO1) and in mitochondrial biogenesis. MiR‐4433b‐5p also targets PIK3R1, the gene that encodes for the p85alpha regulatory subunit of PI3K (Table S6‐A‐B). The other identified target mRNAs are presented in the Supporting Information results and in Table S6.

### 3.5 Correlation Between microRNAs Differential LEs and Clinico‐Biological Parameters Changes (Table 4)

**TABLE 4:** The associations between microRNA differential levels of expression and the physiological and biochemical variables in participants following 12‐weeksHIIT with placebo or citrulline supplementation.

A: Muscle biopsy											
**miR‐504‐5p**											
ALL (*n =* 28)				CIT (*n =* 14)				MEN (*n =* 17)			
Parameter	Correlation (r)	*p*	p‐holm	Parameter	Correlation (r)	*p*	p‐holm	Parameter	Correlation (r)	*p*	p‐holm
BW	+0.36	**	*	Hip circumference	+0.59	*	ns	Appendicular FM	+0.49	*	ns
Waist circumference	+0.37	**	*	ChairST	+0.59	*	ns	Hip circumference	+0.55	*	ns
Ferritin	+0.38	**	*	Takai index	−0.56	*	ns	6mWT	−0.63	*	ns
Hip circumference	+0.32	*	ns	Hip TScore	−0.77	*	ns				
Thigh circumference	+0.29	*	ns	IGF‐1	−0.60	*	ns				
Android FM	+0.28	*	ns	AT‐DGAT1	+0.63	*	ns				
Non HDL‐chol	+0.27	*	ns	AT‐UCP1	+0.74	*	ns				
**miR‐744‐5p**				**miR‐151a‐3p**							
DYNAPENIC (*n* = 21)				MEN (*n =* 17)				> 65 y.o (*n =* 23)			
Parameter	Correlation (r)	*p*	p‐holm	Parameter	Correlation (r)	*p*	p‐holm	Parameter	Correlation (*r*)	*p*	p‐holm
Trunk FM	+0.55	**	*	4mWTf	+0.48	*	ns	TG	+0.45	*	*
Android FM	+0.63	**	ns	4mWT speed	−0.55	*	ns				
Legs LM	+0.51	*	ns	Insulin	+0.60	*	*				
Gynoid LM	+0.52	*	ns	HOMA‐IR	+0.57	*	ns				
Appendicular LM	+0.44	*	ns								
**miR‐127‐5p**											
BMI < 30 (*n* = 16)											
Parameter	Correlation (*r*)	*p*	p‐holm								
4mWT speed	+0.52	*	ns								

B: Blood samples											
**miR‐151a‐3p**											
All (*n =* 68)				WOMEN (*n =* 36)				CIT (*n =* 37)			
Parameter	Correlation (*r*)	*p*	p‐holm	Parameter	Correlation (*r*)	*p*	p‐holm	Parameter	Correlation (*r*)	*p*	p‐holm
TUGf	−0.26	**	*	ChairST	−0.38	*	ns	Adiponectin/Leptin	−0.41	*	*
4mWT	−0.24	**	*	Takai index	+0.39	*	ns	Diastolic AP	−0.37	*	*
4mWT speed	+0.24	**	*	Leptin	−0.36	*	ns	< 65 y.o (*n* = 24)			
4mWTf	−0.24	**	*	> 65 y.o (*n* = 44)				4mWT dble Task	−0.46	*	ns
4mWTf speed	+0.23	**	*	Legs max strength	+0.36	*	ns	4mWT dble Task speed	+0.45	*	ns
4mWT dble task	−0.23	**	*	Equ YF	+0.54	*	ns	BMI < 30 (*n =* 16)			
4mWT dble task speed	+0.25	**	*	Diastolic AP	−0.37	*	ns	ChairST	−0.35	*	ns
VO2peak	+0.27	**	*	nonHDL chol	+0.33	*	ns	Takai index	+0.37	*	ns
ChairST	−0.21	*	*	DYNAPENIC (*n =* 45)							
HDL	0.24	**	*	Adiponectin/Leptin	−0.41	*	*				
**miR‐106b‐5p**								**miR‐484**			
PLA (*n =* 31)				< 65 y.o (*n =* 24)				PLA (*n =* 31)			
Parameter	Correlation (r)	*p*	p‐holm	Parameter	Correlation (r)	*p*	p‐holm	Parameter	Correlation (r)	*p*	p‐holm
4mWTf	+0.41	*	ns	4mWTf	+0.43	*	ns	ChairST	+0.37	*	ns
4mWT speed	−0.40	*	ns	4mWTf speed	−0.45	*	ns	Takai index	−0.40	*	ns
nonHDL‐chol	−0.37	*	ns	Adiponectin	−0.48	**	ns	Adiponectin	−0.47	**	*
				NEFA	+0.41	*	ns				
**miR‐4433b‐5p**								**miR‐483‐3p**			
ALL (*n =* 68)				WOMEN (*n =* 36)				BMI < 30 (*n =* 16)			
Parameter	Correlation (r)	*p*	p‐holm	Parameter	Correlation (r)	*p*	p‐holm	Parameter	Correlation (r)	*p*	p‐holm
Appendicular FM %	+0.20	*	ns	Total FM	+0.43	*	ns	Arms LM	−0.45	*	*
Gynoid FM%	+0.23	*	ns	Total FM%	+0.45	**	*				
Legs FM %	+0.24	*	ns	Arms FM%	+0.55	***	**				
Total LM	−0.24	*	ns	Appendicular FM	+0.37	*	ns				
Arms LM	−0.21	*	ns	Trunk FM%	+0.40	*	ns				
Gynoid LM	−0.21	*	ns	Android FM%	+0.38	*	ns				
Legs FM	−0.20	*	ns	Hip circumference	+0.41	*	ns				
Appendicular LM	−0.19	*	ns	Total LM	−0.34	*	ns				
Trunk LM	−0.25	**	ns	4mWTf speed	+0.37	*	ns				
Step test	−0,33	***	*	>65 y.o (*n* *=* 44)							
4mWT	+0.24	*	ns	Arms LM	−0.37	*	*				
4mWT speed	−0.22	*	ns	AT‐UCP1	0.45	*	*				
4mWT dble task	+0.34	***	**								
4mWT dble task speed	−0.33	***	*								
TUG	+0.23	*	ns								
TUGf	0.24	*	ns								
Legs max strength	−0.22	*	ns								
Legs max strength/BW	−0.21	*	ns								
Takai index	−0.20	*	ns								
Arms BMC	−0.21	*	ns								
AT PPARγ	+0.35	**	ns								
AT LPL	+0.26	*	ns								
AT ATGL	+0.31	*	ns								
AT PLIN3	+0.27	*	ns								

*Note:* The differential level of expression of microRNAs in muscle biopsy (4A) and in serum (4B) was correlated with delta changes of clinical and biological variables outcomes (%), before and after 12‐week HIIT. Correlations parameters were done by a Spearman correlation test (*p‐value ≤ 0.05; **p‐value ≤ 0.01; ***p‐value ≤ 0.001). P‐holm corresponds to *p*‐values adjusted for multiple comparisons using the Holm step‐down procedure. ALL, CIT, men, women, > / < 65 years‐old, BMI</>30 and dynapenic represent the subset of participants, according to sex, age, body mass index, supplemented with L‐citrulline (CIT) or with placebo (PLA) and for all participants (ALL: HIIT‐PLA and HIIT‐CIT combined), following 12‐week HIIT. Anthropometric parameters: BW: body weight; BMI: body mass index; FM and LM: fat mass and bone‐free lean mass measured by dual‐energy X‐ray absorptiometry; circumference; muscle strength, indices and power were recorded as limb muscle strength and by the Takai test. Functional and aerobic capacities were recorded as TUG normal and fast (f): Timed Up and Go (s), 4mWTn, f: 4‐m walking test normal and fast (s) and speed (m/s), 6mWT; 6‐min walking test (m), step test (n), chairST: chair stand test (s), VO2 peak (ml/kg/min). Biological markers: insulin, leptin, adiponectin, ferritin concentration were measured by Elisa [8]. HOMA‐IR: homeostasis model of assessment of insulin resistance index, IGF: insulin growth factor‐1, SBP, DBP: systolic and diastolic blood pressure. Adipocyte tissue (AT) and lipid metabolism outcomes were assessed by measuring the expression level of genes selected for representing important processes expected to be involved in AT metabolism and by blood profiling assay [8]: adipocyte browning (uncoupling protein‐1a [UCP1‐a]), adipocyte differentiation: (PPARgamma2 transcriptional regulation of adipocyte metabolism [PPARγ2]), (peroxisome proliferator‐activated receptor‐gamma coactivator‐1alpha [PGC1‐α]); lipolysis: (hormone sensitive lipase [HSL]), nonesterified free fatty acid (NEFA), adipose triglyceride lipase (ATGL); lipid intake, transport and oxidation: (triglyceride [TG]), cholesterol (chol), lipoprotein lipase (LPL), lipid droplet homeostasis: perilipin1 (PLIN1), lipogenesis: diacylglycerol acyl transferase1/2 (DGAT2). y.o: years old.

In muscle biopsies, miR‐504‐5p downregulation showed significant associations with BW reductions (*r* = 0.36, *p* < 0.01), waist circumference and ferritin levels (*r* = 0.38, *p* < 0.01 < 0.05 after Holm correction). In HIIT‐CIT, miR‐504‐5p was associated with a lower hip circumference, expression of adipocyte metabolism genes (AT‐UCP1, AT‐DGAT2) and improved physical performance (chair‐to‐stand test [ChairST], Takai index) and to a higher IGF‐1 level (*r* = −0.60, *p* < 0.05) which did not remain significantly correlated after Holm correction. Additionally, in men, lower appendicular FM and better 6‐min walking test (6 mWT) were significantly correlated with miR‐504‐5p expression. The decrease in muscle miR‐151a‐3p postintervention correlated with improved triglyceride levels in participants > 65 years (*r =* 0.45, *p* < 0.05, < 0.05 after Holm correction) and reduced insulin levels (*r =* 0.60, *p* < 0.05, < 0.05 after Holm correction) and HOMA‐IR in men, with improved 4‐m walking test fast (4mWTf) performance and speed (4mWTf, *r =* 0.48, *p* < 0.05). In dynapenic participants, miR‐744‐5p upregulation correlated with LM gains (*r =* 0.50, *p* < 0.05). The miR‐127‐5p upregulation in participants with a BMI < 30 was associated with improved 4mWTspeed (*r =* 0.52, *p* < 0.05).

In serum, the increase of miR‐151a‐3p was associated with improvements in functional capacity measures, including the performance at the Timed Up and Go (TUG), 4mWTspeed, chairST and aerobic capacity (6mWT, V02peak, *r =* 0.27, *p* < 0.01 < 0.05 after Holm correction). It was associated with subgroup‐specific correlations: participants < 65 years showed faster 4mWTspeed, while participants > 65 years old showed greater leg strength, associated with lower leptin levels (*r =* −0.36, *p* < 0.05) in women.

The miR‐106b‐5p downregulation was associated with improvements in 4mWTf (*p* < 0.05) along with higher adiponectin levels and non‐esterified fatty acids (NEFA) decrease, particularly in younger participants (< 65 years old) and in HIIT‐PLA. The miR‐484 downregulation in HITT‐PLA was associated to a gain of performance capacity (chairST and Takai index, *p* < 0.05) and to a higher adiponectin level (*r =* −0.47, *p* < 0.01, < 0.05 after Holm correction).

HIIT‐induced downregulation of circulating miR‐4433b‐5p was associated with significant FM reduction along with the LM gain in HIIT‐ALL, with a marked effect in women (total FM%, *r =* 0.45, *p* = 0.01, < 0.05 after Holm correction; total LM, *r =* −0.34, *p* < 0.05). This regulation was also associated with score improvement of functional tests (step test *r =* −0.33, *p* < 0.001, < 0.05 after Holm correction), muscle power (Takai index, *r =* −0.20, *p* < 0.05) and with the decrease activity of some AT enzymes (ATpparγ, *r =* 0.35, *p* < 0.01).

The miR‐483‐3p downregulation in participants with lower BMI correlated with improved arm LM (*r =* −0.45, *p* < 0.05, < 0.05 after Holm correction).

## 4 Discussion

Considering the microRNAs' regulatory role in the molecular response to exercise and the correlation of their release from tissues with clinic‐biological parameters changes, microRNAs have been proposed as biomarkers for exercise‐biological performance [17]. By analysing microRNAs' expression in both muscle biopsies and sera of obese older adults, we found 10 microRNAs (miR‐151a‐3p, ‐504‐5p, ‐106a‐5p, ‐744‐5p, ‐483‐3p, ‐484, ‐146b‐5p, ‐127‐5p, ‐136‐3p, ‐4433b‐5p) that were significantly differentially expressed following a 12‐week HIIT with or without CIT supplementation. We also highlighted that this microRNA regulation appears differently modulated according to sex, age, BMI or muscle strength status (dynapenia).

Supplementing HIIT with CIT induced a decrease of muscle miR‐504‐5p without any other significant changes in the microRNA differential LEs.

### 4.1 Lack of Myo‐ and Muscle‐Related microRNAs Response to Training

By analysing the muscle miRNome, we found that the expression of the muscle‐specific microRNAs that include the myo‐microRNAs (miR‐133a, b, miR‐1, miR‐206) and the muscle‐related microRNAs (miR‐208a, ‐b, ‐499) was not modulated in HIIT‐PLA or HIIT‐CIT. The miR‐133a‐3p significant downregulation in the discovery phase was not further validated in the larger population. This muscle‐specific microRNA response to exercise and nutritional interventions greatly depends on the type, mode, duration of intervention as well as the metabolic status of participants. In response to resistance training, miR‐1, miR‐133 and miR‐206 are downregulated, whereas endurance training or acute anabolic stimulus induces an upregulation or no change in their expression [18, 19, 20]. The muscle‐specific microRNAs response to HIIT also differed from what has been reported following resistance or aerobic training either in young or older participants [21, 22]. Moreover, age and metabolic impairment‐induced alterations of these microRNAs' expression are associated with impaired skeletal muscle adaptations to exercise‐mediated anabolic stimulation and to blunted microRNAs response [11, 23, 24]. Our data suggest that the lack of response of these skeletal muscle microRNAs to HIIT stimulation and CIT supply depends on age and metabolic status which are known to induce anabolic resistance [10, 23].

In contrast, besides the myo‐microRNAs and the muscle‐related microRNAs, several unspecific microRNAs which are excreted by various tissues and cell types were altered in both muscle biopsies and blood samples post‐intervention. Exercise releases microRNAs actively and passively from muscles and other tissues, affecting their circulating levels [17]. They serve as intercellular messengers, inducing transcriptomic changes in distant tissues. Specific exosomal microRNAs play a crucial role in maintaining glucose homeostasis and insulin sensitivity [25, 26]. In response to exercise, specific microRNAs are released in circulating exosomes and regulate metabolic pathways in distant tissues, impacting whole‐body metabolism [27, 28]. These previous studies have suggested that HIIT improvement of functional performance, aerobic capacity, adiposity profiles [8] [S2 and S3] and glycemia [29] in participants of our study might be partly mediated by in situ or released microRNAs, impacting protein anabolism, lipid and carbohydrate metabolism.

### 4.2 Muscle Biopsy and Circulating microRNAs as Potential Biomarkers of HIIT‐Induced Beneficial Effects on Body Composition, Muscle Power and Functional Capacity

Age, adiposity and muscle functional loss trigger aberrant microRNA expression in tissue along with changes in circulating microRNA profile [11, 23]. In the whole heterogenic population of our study, we report that the modulation of microRNAs LE following intervention differs in participants according to sex, age, dynapenic status and obesity and is differently associated with body composition, muscle strength and functional capacities improvements in HIIT‐PLA, HIIT‐CIT and in the participants' subsets.

#### 4.2.1 In Muscle Biopsy

The decrease in miR‐504‐5p LE in HIIT‐ALL, HIIT‐CIT and men correlated with lower BW and FM along with a better muscle power and functional capacity scores. This microRNA, expressed in cardiac and vascular smooth muscle cells [30], is upregulated in response to high glucose, palmitic acid and diabetes, which also induces inflammatory genes while decreasing the expression of growth factor genes (e.g., early growth response protein 2 [Egr2], growth factor receptor‐bound protein 10 [Grb10]) and contractile genes [31]. It is co‐transcriptionally expressed with fibroblast growth factor 13 (FGF13), an inhibitor of the ribosomal transcription, which attenuates protein synthesis [31]. In HIIT‐CIT, the increase of physical performance and of serum IGF‐1 level indicated the stimulation of protein anabolism that was negatively correlated with the miR‐504‐5p downregulation. The modulation of miR‐504‐5p LE and its association with beneficial effects in clinical adaptations in participants supplemented with CIT but not in HIIT‐PLA suggests that the muscle miR‐504‐5p downregulation may stimulate protein anabolism under CIT supplementation. The circulating miR‐504‐5p was undetectable probably because it is mostly expressed in vascular smooth muscle cells and not released by others tissues [30]. This limits its biomarker ability to biopsy of skeletal muscle.

The miR‐744‐5p upregulation, observed only in dynapenic participants, was positively correlated with LM indicating an adaptive response in muscle recovery and strength that has been reported in the context of muscle wasting and weakness [32]. MiR‐744‐5p expressed in *vastus lateralis* biopsy and proliferative myoblasts is a regulator of myogenesis that has been positively associated with recovery of muscle mass and muscle strength in critical illness survivors with muscle wasting and weakness [32]. Despite the HIIT‐induced miR‐744‐5p upregulation in muscle biopsy, it was downregulated in serum of HIIT‐PLA, but this EL decrease in serum of both dynapenic and nondynapenic groups did not correlate with a gain in muscle power or muscle power ratio. The miR‐744‐5p downregulation has been previously reported in serum of sarcopenic versus healthy subjects without evidence to associate the circulating EL to the sarcopenic processes [33]. The discrepancy between miR‐744‐5p regulation in muscle and serum suggests that it may be released from other tissues and that only miR‐744‐5p in muscle biopsy is a candidate biomarker of intervention.

MiR‐151a‐3p was downregulated in muscle biopsy of men and > 65 years‐old participants correlating with better 4mWT scores and speed, potentially due to its regulatory effects on myosin expression and muscle fibre type. MiR‐151a‐3p reduction is associated with increased ATPase2 activity, which shifts muscle fibre type by upregulating slow MHC‐beta [34]. The association between its downregulation and the triglyceride reduction and improvement in insulin resistance remains ununderstood since it could not be explained by miR‐151a‐3p gene targeting without any report in previous study.

#### 4.2.2 In Serum

The improvement of functional capacities and muscle power score (Takai index) correlated to the miR‐151a‐3p up‐regulation suggests that circulating miR‐151a‐3p might be a biomarker of FAK activation to HIIT response. Indeed, miR‐151a‐3p's significant association with several functional markers was confirmed by Holm procedure, and this microRNA is expressed together with its host gene FAK, encoding focal adhesion kinase [35]. FAK activation supports muscle homeostasis by regulating myoblast development and muscle fibre formation [36], suggesting that circulating miR‐151a‐3p might be a biomarker of FAK activation to HIIT response. However, the differing trends of modulation between muscle and circulating compartments suggest their independence and indicate that miR‐151a‐3p likely originates from non‐muscle tissue, without data in our study suggesting its source.

The decrease of serum miR‐4433b‐5p correlated with FM reductions, LM gains, and could improve functional capacities. Its absence in muscle biopsy and adipose‐specific expression suggests a systemic regulatory role, potentially through IGF‐1 receptor (IGF‐1R) and PIK3R1, the gene that encodes for the p85alpha regulatory subunit of phosphoinositide 3‐kinase (PI3K), improving insulin receptor signaling pathway and glucose homeostasis in obesity enhancing IGF‐1 signaling to promote protein anabolism [37].

MiR‐106b‐5p was downregulated in muscle biopsies of participants with BMI > 30 kg/m^2^, in serum of HIIT‐PLA and of participants under 65 years. Its downregulation was correlated with higher adiponectin, lower NEFA levels and better 4mWT scores. This aligns with studies suggesting that antagonizing miR‐106b‐5p enhances muscle regeneration and insulin sensitivity via GLUT4 and IGF‐1/IGF‐1R pathways and inhibits Ucp1 (a brown adipocyte marker) mRNA improving insulin signalling, supporting reductions in obesity and insulin resistance [38, 39] [S7].

#### 4.2.3 MiR‐4433b‐5p, ‐106b‐5p and ‐483‐3p Downregulation in Response to HIIT May Be Consistent With Protein Anabolic Signalling

The web‐based computational databases identified the miR‐4433b‐5p, ‐106b‐5p and ‐483‐3p interactions with their predicted and previously reported experimentally validated target genes (Supporting Information results). We found that the miR‐4433b‐5p, ‐106b‐5p and ‐483‐3p, which form part of the HIIT response, target the IGF‐1/IGF‐1‐R/−R2 and PI3K/AKT pathways; thereby, they may act together by influencing muscle protein synthesis, degradation and metabolic homeostasis.

For example, taking into account *in silico* results, the decrease of miR‐4433b‐5p level may induce a potential upregulation of the IGF‐1/IGF‐1R and PI3K signalling, leading to the activation of the downstream AKT/mTOR pathway which is essential for protein synthesis and muscle homeostasis. IGF‐1 binding to IGF‐1R activates AKT. Activation of AKT inhibits FoxO transcription factors, reducing the expression of genes involved in protein degradation, such as atrogin‐1 and MuRF1, thereby decreasing muscle protein breakdown. Concurrently, AKT activation stimulates mTORC1, promoting protein synthesis through the phosphorylation of key regulators like p70S6 kinase (S6K) and eukaryotic initiation factor 4E‐binding protein 1 (4E‐BP1) [30, 31]. This results in increased muscle protein synthesis and hypertrophy, contributing to improvements in muscle mass and muscle strength. Furthermore, AKT activation stimulates mTORC2, leading to additional phosphorylation events involving AKT, S6K and translation factors eIF4E and eIF2B, thereby further enhancing protein synthesis [10]. The decrease of miR‐4433b‐5p, ‐106b‐5p and ‐483‐3p would be predictive to the enhancing of the IGF‐1/IGF‐1R signalling that might promote protein anabolism and impact metabolic pathways related to muscle function and energy metabolism. While these associations and *in silico* results are consistent with an anabolic‐favouring milieu, we did not measure direct molecular markers of protein anabolic signalling (e.g., AKT/mTOR phosphorylation and myofibrillar protein synthesis). Therefore, these findings should be regarded as associational and hypothesis‐generating, warranting confirmation with targeted molecular assays.

### 4.3 Modulation of microRNAs Response to CIT Supplementation

Our previous studies report that CIT supplementation induces greater improvements in upper muscle strength and walking speed than HIIT alone without increasing the beneficial effect of HITT on VO2 max [S2]. However, HIIT combined with CIT supplementation induced weak modifications of microRNA differential LEs compared to HIIT alone. Indeed, only miR‐136‐3p LE was significantly different between HIIT‐PLA and HIIT‐CIT at T12. The main effect of adding a CIT supplementation to HIIT is the strong miR‐504‐5p downregulation in muscle whereas a tendency to upregulation was observed in HIIT‐PLA. This miR‐504‐5p modulation in HIIT‐CIT was significantly associated with increases in circulating IGF‐1 level, muscle strength and functional capacity, underscoring that miR‐504‐5p may be important in enhancing CIT's efficacy through the mTOR pathway [40]. These associations did not remain significant after Holm correction and remain hypothetic since they were reported only in 16 participants.

It also fits with the fact that adding CIT to HIIT stimulated the decrease of circulating miR‐4433b‐5p compared to HIIT alone which may result in a higher mTOR pathway activation [40]. The miR‐146b‐5p upregulation in muscle and the downregulation of circulating miR‐136a‐3p, at the end of intervention, might contribute to the CIT beneficial effect on LM and muscle strength. Indeed miR‐146b‐5p promotes myoblast differentiation and proliferation, which may contribute to the beneficial effect of CIT on muscle, as well as to the protective effect against atherosclerosis [41].

### 4.4 Strengths and Limitations

We profiled muscle and circulating microRNAs in participants with documented lifestyle and diet. Contrary to expectations, circulating microRNAs did not fully mirror biopsy changes, suggesting contributions from nonmuscle tissues, notably adipose [25, 26, 27, 28]. Adipose‐derived exosomal microRNAs, which regulate insulin sensitivity and metabolism, may help explain systemic training adaptations and serve as potential biomarkers in older adults with obesity [26].

Participants were relatively healthy, community‐dwelling older adults with obesity and limited comorbidities. As such, the external validity of our findings to clinically complex populations (e.g., sarcopenia, frailty, multimorbidity or chronic disease) is limited. The discovery NGS subset was small (*n* = 13), increasing the risk of both false positives and false negatives; we mitigated alpha risk by controlling the false discovery rate in discovery and by validating prespecified candidates by TaqMan RT‐qPCR in the full cohort. Because metabolic profiles varied, we conducted prespecified subgroup analyses (sex, age, BMI and dynapenia), which reduced sample sizes and statistical power; to limit underpower, we also analysed the combined HIIT cohort (HIIT‐ALL). The pre–post design may have missed early or transient responses [S8], and the limited number of biopsies and multiple subgroup tests increase statistical error. As a secondary analysis of a randomized trial, the study lacked a nonexercise control and a CIT‐only arm, precluding clear separation of training from time effects and CIT effects independent of HIIT. Although sampling was standardized and participants were instructed to maintain usual habits, these measures cannot substitute for a control. The absence of device‐logged session power/work precludes formal dose–response analyses; dedicated future work could be warranted. Finally, because the serum miRNome reflects signals from multiple tissues, adipose‐specific profiling is warranted. Overall, the patterns of microRNA modulation with HIIT and CIT—and the observed sex‐ and age‐specific differences—appear robust in the full cohort. By contrast, BMI‐stratified findings and all association analyses should be interpreted cautiously given small subgroup sizes and the number of parameters. To aid balanced interpretation, we applied a conservative Holm step‐down correction which reduces power and requires cautious interpretation. Significant associations are consistent with prior literature but remain observational and do not establish causality; we did not assess direct molecular readouts of anabolic signalling (e.g., AKT/mTOR phosphorylation or myofibrillar protein synthesis), which limits mechanistic inference.

## 5 Conclusion

In this secondary, exploratory analysis of a randomized, double‐blind trial, HIIT with or without CIT supplementation did not alter the myo‐microRNAs and muscle‐related microRNAs LE but modulated the unspecific microRNAs in muscle biopsy and serum of obese older adults. Adding CIT to HIIT induced a downregulation of muscle miR‐504‐5p versus HIIT alone. Changes of miR‐151a‐3p, miR‐4433b‐5p, miR‐504‐5p, miR‐483‐3p, miR‐106b‐5p and miR‐744‐5p LE in response to interventions might be associated with beneficial outcomes in body composition, functional capacity and metabolic markers, depending on participants' sex, age, BMI and dynapenic status. These results provide insights into the molecular mechanisms regulating the HIIT effects and suggest that these microRNAs are potential candidate biomarkers of personalized training adaptation. These microRNAs should be regarded as candidate biomarkers of HIIT response; their clinical utility and mechanistic relevance warrant replication in independent cohorts and confirmation with direct molecular readouts and nonexercise controls.

## Ethics Statement

The authors certify that they comply with the ethical guidelines for publishing in the *Journal of Cachexia, Sarcopenia and Muscle*.

## Conflicts of Interest

The authors declare no conflicts of interest.

## Supporting information


**Table S1:** The participant characteristics at baseline. BMI: body mass index; LM: lean mass; FM: fat mass; MoCA: Validated Montreal Cognitive Assessment; METs: metabolic equivalent of task.
**Table S2:**. Identification of TaqMan advanced microRNAs assays of the spike quality control, of the three endogenous normalizers and of the candidate microRNAs used for the RT‐qPCR analysis of the nineteen microRNAs in the validation phase. All the nomenclature is according to miRBase V21 and the TaqMan Advanced microRNA assays are from Applied Biosystems.
**Table S3:**. The expression level of myo‐microRNAs and muscle‐related‐microRNAs in muscle biopsy and serum from the participants in HIIT‐PLA and HIIT‐CIT at baseline. The absolute quantification expressed as TMM for each microRNA identified in both muscle and circulating compartment was provided by NGS analysis. The total amount (total TMM) of microRNAs expressed in muscle and in serum has been sum up to calculate the TMM ratio of each myo‐microRNAs (miR‐133, −1, −206) and of muscle‐related‐microRNAs (miR‐208, −499, −486) to the total microRNAs expressed in muscle and in serum. Regulations of expression and functions are provided for each microRNA (↑,↓) indicates the increase or decrease of expression, function, respectively.
**Table S4:**. The screening phase by NGS analysis for comparison of microRNAs differential expression in the participants supplemented with placebo before and after 12‐weeks HIIT and in the participants supplemented with L‐citrulline before and after 12‐weeks HIIT. The absolute microRNAs amount in the participants supplemented with placebo before and after 12‐ weeksHIIT (PLA) and in the participants supplemented with L‐citrulline (CIT) before and after 12‐weeks HIIT was reported as TMM. The Log_2_ FC and the comparison between groups (*p <* 0.05) are indicated.
**Table S5:**. The selection of the 19 microRNAs for further analysis in the validation phase by Real‐Time quantitative PCR. The 19 microRNAs were selected on the basis of their highly significant differential expression and on their high NGS reads between muscle biopsy or serum of the HIIT‐PLA (PLA) and HIIT‐CIT (CIT) groups. The absolute amount (TMM) and the Log_2_ FC for each microRNA are indicated.
**Table S6:** The interaction of microRNAs with their metabolic and signalling pathway‐related genes in dynapenic‐obese elderly following 12‐weeks HIIT. (A) We used the Diana MiRTarBase V8 reporting experimentally validated target genes (Diana MiRTarBase V8) and MiRWalk V3, http://multimir.org predicting target genes to search for the potential interactions of the microRNA seed region with genes that might be involved in response to HIIT and L‐citrulline supplementation. We have also mined the literature for experimentally validated microRNA targets to determine which molecular pathways would be likely altered in response to exercise and found that differentially regulated microRNAs target the gene‐related to skeletal muscle development and regeneration, myogenesis, mitochondrial biogenesis, ROS production, glucose and lipid metabolism, protein anabolism, adipogenesis and inflammation. (B) We used TargetScanHuman, release7.2 to report predictive target genes (3’UTR). Mer indicates the number of exact nucleotide match to positions of the mature microRNA (the seed) with the 3’UTR; Predicted efficacy of targeting are calculated as cumulative weighted context++ scores of the sites and PCT indicates the probability of conserved targeting.
**Table S7:** The expression level of microRNAs analysed by RT‐qPCR in muscle biopsy and serum from the participants in HIIT‐ALL (ALL), HIIT‐PLA (PLA), HIIT‐CIT (CIT) and participant subsets (men, women, > / < 65 years‐old, BMI < / > 30 and dynapenic or not dynapenic, in muscle (S7A) and serum (S7B) (*p‐value ≤ 0.05; **p‐value ≤ 0.01; ***p‐value ≤ 0.001). The results are presented as the median of Log2 fold change. Empty cells correspond to microRNAs that are not quantifiable in a specific compartment by RT‐qPCR. The *p*‐values were calculated using the Wilcoxon matched pairs signed rank test, except for the DLE and T12 calculated using the Mann–Whitney test. BMI: body mass index; y.o: years; DLE: difference level of expression; *P*: *p*‐value.
