# Supplementary material for: Skeletal Muscle and Circulating microRNAs Adaptations to 12‐Week HIIT With or Without L‐Citrulline in Obese Older Adults
Source: J Cachexia Sarcopenia Muscle. 2026 Apr 3;17(2):e70267. doi: 10.1002/jcsm.70267 (PMC13051928; doi:10.1002/jcsm.70267)
Supplement: Supplementary file 1 — Table S1: The participant characteristics at baseline. BMI: body mass index; LM: lean mass; FM: fat mass; MoCA: Validated Montreal Cognitive Assessment; METs: metabolic equivalent of task. Table S2:. Identification of TaqMan advanced microRNAs assays of the spike quality control, of the three endogenous normalizers and of the candidate microRNAs used for the RT‐qPCR analysis of the nineteen microRNAs in the validation phase. All the nomenclature is according to miRBase V21 and the TaqMan Advanced microRNA assays are from Applied Biosystems. Table S3:. The expression level of myo‐microRNAs and muscle‐related‐microRNAs in muscle biopsy and serum from the participants in HIIT‐PLA and HIIT‐CIT at baseline. The absolute quantification expressed as TMM for each microRNA identified in both muscle and circulating compartment was provided by NGS analysis. The total amount (total TMM) of microRNAs expressed in muscle and in serum has been sum up to calculate the TMM ratio of each myo‐microRNAs (miR‐133, −1, −206) and of muscle‐related‐microRNAs (miR‐208, −499, −486) to the total microRNAs expressed in muscle and in serum. Regulations of expression and functions are provided for each microRNA (↑,↓) indicates the increase or decrease of expression, function, respectively. Table S4:. The screening phase by NGS analysis for comparison of microRNAs differential expression in the participants supplemented with placebo before and after 12‐weeks HIIT and in the participants supplemented with L‐citrulline before and after 12‐weeks HIIT. The absolute microRNAs amount in the participants supplemented with placebo before and after 12‐ weeksHIIT (PLA) and in the participants supplemented with L‐citrulline (CIT) before and after 12‐weeks HIIT was reported as TMM. The Log2 FC and the comparison between groups (p < 0.05) are indicated. Table S5:. The selection of the 19 microRNAs for further analysis in the validation phase by Real‐Time quantitative PCR. The 19 microRNAs were selected on the [file JCSM-17-e70267-s001.docx]

**Supplementary Table Legends**

**Table S1**. The participant characteristics at baseline.

*BMI: body mass index; LM: lean mass; FM: fat mass; MoCA: Validated Montreal Cognitive Assessment; METs: metabolic equivalent of task.*

**Table S2**. Identification of TaqMan advanced microRNAs assays of the spike quality control, of the three endogenous normalizers and of the candidate microRNAs used for the RT-qPCR analysis of the nineteen microRNAs in the validation phase. *All the nomenclature is according to miRBase V21 and the TaqMan^™^ Advanced microRNA assays are from Applied Biosystems.*

**Table S3**. The expression level of myo-microRNAs and muscle-related-microRNAs in muscle biopsy and serum from the participants in HIIT-PLA and HIIT-CIT at baseline.

*The absolute quantification expressed as TMM for each microRNA identified in both muscle and circulating compartment was provided by NGS analysis. The total amount (total TMM) of microRNAs expressed in muscle and in serum has been sum up to calculate the TMM ratio of each myo-microRNAs (miR-133, -1, -206) and of muscle-related-microRNAs (miR-208, -499, -486) to the total microRNAs expressed in muscle and in serum.* *Regulations of expression and functions are provided for each microRNA (↑,↓) indicates the increase or decrease of expression, function, respectively.*

**Table S4**. The screening phase by NGS analysis for comparison of microRNAs differential expression in the participants supplemented with placebo before and after 12-weeks HIIT and in the participants supplemented with L-citrulline before and after 12-weeks HIIT.

*The absolute microRNAs amount in the participants supplemented with placebo before and after 12- weeksHIIT (PLA) and in the participants supplemented with L-citrulline (CIT) before and after 12-weeks HIIT was reported as TMM. The Log_2_ FC and the comparison between groups (p<0.05) are indicated.*

**Table S5**. The selection of the nineteen microRNAs for further analysis in the validation phase by Real-Time quantitative PCR.

*The nineteen microRNAs were selected on the basis of their highly significant differential expression and on their high NGS reads between muscle biopsy or serum of the HIIT-PLA (PLA) and HIIT-CIT (CIT) groups. The absolute amount (TMM) and the Log_2_ FC for each microRNA are indicated.*

**Table S6.** The interaction of microRNAs with their metabolic and signaling pathway-related genes in dynapenic-obese elderly following 12-weeks HIIT.

***(A****) We used the Diana MiRTarBase V8 reporting experimentally validated target genes (Diana MiRTarBase V8) and MiRWalk V3,* [***http://multimir.org***](http://multimir.org/) *predicting target genes to search for the potential interactions of the microRNA seed region with genes that might be involved in response to HIIT and L-citrulline supplementation. We have also mined the literature for experimentally validated microRNA targets to determine which molecular pathways would be likely altered in response to exercise and found that differentially regulated microRNAs target the gene-related to skeletal muscle development and regeneration, myogenesis, mitochondrial biogenesis, ROS production, glucose and lipid metabolism, protein anabolism, adipogenesis and inflammation.*

*(B) We used TargetScanHuman, release7.2 to report predictive target genes (3’UTR). Mer indicates the number of exact nucleotide match to positions of the mature microRNA (the seed) with the 3’UTR; Predicted efficacy of targeting are calculated as cumulative weighted*[*context++ scores*](https://www.targetscan.org/vert_70/docs/context_score.html)*of the sites and* [*P_CT_*](https://www.targetscan.org/docs/pct.html) *indicates the probability of conserved targeting.*

**Table S7.** The expression level of microRNAs analyzed by RT-qPCR in muscle biopsy and serum from the participants in HIIT-ALL (ALL), HIIT-PLA (PLA), HIIT-CIT (CIT) and participant subsets (men, women, >/<65 years-old, BMI</>30 and dynapenic or not dynapenic, in muscle (S7A) and serum (S7B) *(* p-value ≤ 0.05; ** p-value ≤ 0.01; *** p-value ≤ 0.001)*

*The results are presented as the median of Log2 fold change. Empty cells correspond to microRNAs that are not quantifiable in a specific compartment by RT-qPCR. The p-values were calculated using the Wilcoxon matched pairs signed rank test, except for the DLE and T12 calculated using the Mann-Whitney test.*

*BMI: body mass index; y.o: years; DLE: difference level of expression; P: p-value*

**Table S1**

| **Variables** | **Total Population Sub-Sample (NGS)**  **(n=68) (n=13)** |
| --- | --- |
| Age (years) | 67±4 66.7±4.8 |
| Sex (% of men) | 47 % (32/68) 0 (0) |
| MoCa (x/30) | 27.5±1.7 27.1±1.9 |
| SF-36 (Total ; /100) | 79.4±14.6 80.7±16.4 |
| Body Weight (BW ; kg) | 80.7±13.5 87.4±5.8 |
| BMI (kg/m²) | 29.6±4.8 30.1±2.4 |
| Waist circumference (cm) | 104.4±12.7 104.5±14.1 |
| Total LM (kg) | 47.1±8.6 52.9±3.9 |
| Total FM (%) | 37.8±7. 3.4 35.3±1 |
| Metabolic syndrome (Yes, %) | 29/62 9/69 |
| MDx Hypertension (Yes; %) | 22/63 8/61.5 |
| MDx Type 2 Diabetes (Yes; %) | 8/63 3/23 |
| MDx Cholesterol (Yes; %) | 15/63 3/23 |
| Number of steps (n/day) | 5877±3263 5490±3885 |
| METs | 1.17±0.18 1.17±0.22 |

**Table S2**

| **miR Base ID** | **miRBase Accession Number** | **TaqMan Advanced microRNA Assay (ID)** | **Sequence of the mature microRNA**  **5’—————————3’** |
| --- | --- | --- | --- |
| cel-39-3p | MIMAT0000010 | 478293_mir | UCACCGGGUGUAAAUCAGCUUG |
| hsa-191-5p | MIMAT0000440 | 477952_mir | CAACGGAAUCCCAAAAGCAGCUG |
| hsa-222-3p | MIMAT0000279 | 477982_mir | AGCUACAUCUGGCUACUGGGU |
| hsa-361-5p | MIMAT0000703 | 478056_mir | UUAUCAGAAUCUCCAGGGGUAC |
| hsa-127-5p | MIMAT0004604 | 477891_mir | CUGAAGCUCAGAGGGCUCUGAU |
| hsa-136-3p | MIMAT0004606 | 477902_mir | CAUCAUCGUCUCAAAUGAGUCU |
| hsa-151a-3p | MIMAT0000757 | 477919_mir | CUAGACUGAAGCUCCUUGAGG |
| hsa-369-3p | MIMAT0000721 | 478067_mir | AAUAAUACAUGGUUGAUCUUU |
| hsa-483-3p | MIMAT0002173 | 478122_mir | UCACUCCUCUCCUCCCGUCUU |
| hsa-504-5p | MIMAT0002875 | 478144_mir | AGACCCUGGUCUGCACUCUAUC |
| hsa-515-5p | MIMAT0002826 | 478147_mir | UUCUCCAAAAGAAAGCACUUUCUG |
| hsa-625-3p | MIMAT0004808 | 478179_mir | GACUAUAGAACUUUCCCCCUCA |
| hsa-744-5p | MIMAT0004945 | 478200_mir | UGCGGGGCUAGGGCUAACAGCA |
| hsa-136-5p | MIMAT0000448 | 478307_mir | ACUCCAUUUGUUUUGAUGAUGGA |
| hsa-484 | MIMAT0002174 | 478308_mir | UCAGGCUCAGUCCCCUCCCGAU |
| hsa-106b-5p | MIMAT0000680 | 478412_mir | UAAAGUGCUGACAGUGCAGAU |
| hsa-483-5p | MIMAT0004761 | 478432_mir | AAGACGGGAGGAAAGAAGGGAG |
| hsa-133a-3p | MIMAT0000427 | 478511_mir | UUUGGUCCCCUUCAACCAGCUG |
| hsa-516a-5p | MIMAT0004770 | 478978_mir | UUCUCGAGGAAAGAAGCACUUUC |
| hsa-181a-3p | MIMAT0000270 | 479405_mir | ACCAUCGACCGUUGAUUGUACC |
| hsa-4433b-5p | MIMAT0030413 | 479803_mir | AUGUCCCACCCCCACUCCUGU |
| hsa-1277-5p | MIMAT0022724 | 480875_mir | AAAUAUAUAUAUAUAUGUACGUAU |
| hsa-146b-5p | MIMAT0002809 | 483144_mir | UGAGAACUGAAUUCCAUAGGCUG |

|  | **Muscle** | | **Serum** | | | **Regulation of expression/function** | **References** |
| --- | --- | --- | --- | --- | --- | --- | --- |
| **MicroRNA** | **T0 PLA (TMM)** | **T0 CIT (TMM)** | | **T0 PLA (TMM)** | **T0 CIT (TMM)** |  |  |
| **Myo-microRNA** | | | | | | | |
| -133a-3p | 251401  (23%) | 247554  (26%) | | 71.0 | 91.5 | Bicistronic miR-133a/-1 cluster encoding  Regulated by MyoD, MRFs, MEF2, SRF  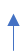myoblast proliferation through the regulation of key genes controlling myogenesis  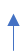muscle regeneration and mitochondrial biogenesis and rate of protein synthesis  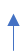 expression in response to acute exercise but 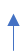after long-term exercise training  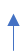 muscle regeneration and mitochondrial biogenesis | PMID: 25553440  PMID: 17008435  PMID: 26708096  PMID: 25460913 PMID: 21606874 |
| -133a-5p | 3944 | 3765 | | nd | nd |  |  |
| -1 | 350643  (32%) | 345532  (37%) | | 63.0 | 73.7 | Regulated by Myogenic regulatory factors, MYoD1, mTORC1, IGF-1  Promotes myoblast differentiation, regeneration  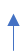rate of protein synthesis | PMID:21030674  PMID: 20724368 PMID: 21606874 PMID: 25553440 |
| -206 | 23477  (2.2%) | 20119  (2.1%) | | 45 | 46 | Bicistronic miR-206/-133b, -1/206 encodings  Regulated by MYOD1  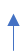satellite stem cell activity 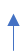fat infiltration  Alter fiber type switching, induces transition from  Proliferation to differentiation | PMID: 1745994  PMID: 26708096  PMID: 18381085 |
| -133b | 2199 | 2257 | | nd | nd | Shares similar targets with miR-133a |  |
|  | | | | | | | |
| **Total TMM** | **1082869** | **936481** | | **1047456** | **861500** |  |  |
|  | | | | | | | |
| **Muscle-related-microRNA** | | | | | | | |
| -208 | 1105 | 1272 | | nd | nd | Encoded in the 3 myosin genes (MYH7)  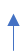by estrogen-related receptor γ , essential for enhancing slow myosin type1 myofibers, myosin switching, 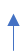muscle growth | PMID: 20965416  PMID: 19922871  PMID: 19828686 |
| -499a-5p | 5521 | 5186 | | 3.3 | 3.1 | Encoded in MYH7B  Regulates muscle fiber shift, muscle growth | PMID: 37371465 PMID: 19922871 PMID: 19828686 |
| -499a-3p | 35 | 30 | | nd | nd |  | PMID: 26708096 |
| -486-5p | 8211  (0.75%) | 6942  (0.74%) | | 293708  (28%) | 172308  (20%) | Encoded in ANK1  Regulated by myoD1, Up-regulated during muscle differentiation, targets Pax7 increasing myoblast differentiation and fusion, regulates PI3-kinase/Akt signaling | PMID: 3258757 PMID: 20142475 |
| -486-3p | 37 | 36 | | 160 | 183 |  |  |

**Table S4**

| **Muscle PLA T12-week vs. T0** | | | | | |
| --- | --- | --- | --- | --- | --- |
| **MicroRNAs** | **PLA_T0 (TMM)** | **PLA_T12 (TMM)** | **Log2 Fold change** | **P value** | **FDR** |
| hsa-miR-133a-3p | 251400.84 | 182131.02 | -0.458898 | 0.00E+00 | 0.00E+00 |
| hsa-miR-516a-5p | 8.06 | 21.96 | 1.56865 | 1.32E-10 | 3.76E-08 |
| hsa-miR-372-3p | 0.46 | 1.81 | 2.18059 | 4.88E-07 | 9.26E-05 |
| hsa-miR-516b-5p | 4.82 | 9.15 | 1.09771 | 1.02E-06 | 1.45E-04 |
| hsa-miR-1283 | 1.4 | 3.45 | 1.33744 | 1.73E-06 | 1.96E-04 |
| hsa-miR-483-5p | 16.68 | 30.21 | 0.897008 | 2.44E-06 | 2.14E-04 |
| hsa-miR-887-3p | 2.12 | 4.65 | 1.19403 | 2.97E-06 | 2.14E-04 |
| hsa-miR-515-5p | 6.3 | 12.22 | 1.00503 | 3.01E-06 | 2.14E-04 |
| hsa-miR-488-3p | 1.1 | 2.56 | 1.17635 | 3.66E-06 | 2.31E-04 |
| hsa-miR-127-5p | 6.62 | 11.06 | 0.737322 | 2.33E-05 | 1.32E-03 |
| hsa-miR-34c-5p | 5.76 | 9.71 | 0.754274 | 2.92E-05 | 1.47E-03 |
| hsa-miR-146b-5p | 58.32 | 115.04 | 0.961523 | 3.09E-05 | 1.47E-03 |
| hsa-miR-504-5p | 23.32 | 37.42 | 0.735304 | 5.71E-05 | 2.50E-03 |
| hsa-miR-539-3p | 1.04 | 2.18 | 1.12185 | 8.51E-05 | 3.38E-03 |
| hsa-miR-889-3p | 3.68 | 6.37 | 0.771989 | 8.92E-05 | 3.38E-03 |
| hsa-miR-181a-3p | 8.4 | 13.43 | 0.700718 | 1.16E-04 | 4.12E-03 |
| hsa-miR-431-5p | 2.75 | 4.83 | 0.791429 | 1.54E-04 | 5.15E-03 |
| hsa-miR-519d-3p | 1.22 | 2.47 | 1.14828 | 1.70E-04 | 5.36E-03 |
| hsa-miR-625-3p | 19.43 | 12.5 | -0.624522 | 1.79E-04 | 5.36E-03 |
| hsa-miR-377-3p | 6.14 | 9.96 | 0.721603 | 2.51E-04 | 7.00E-03 |
| hsa-miR-1277-5p | 10.7 | 18.87 | 1.04113 | 2.58E-04 | 7.00E-03 |
| hsa-miR-136-5p | 11.1 | 18.49 | 0.747121 | 2.75E-04 | 7.12E-03 |
| hsa-miR-136-3p | 18.11 | 28.04 | 0.713488 | 2.93E-04 | 7.24E-03 |
| hsa-miR-181c-5p | 2.33 | 3.81 | 0.708647 | 3.44E-04 | 8.15E-03 |
| hsa-miR-369-3p | 17.82 | 27.13 | 0.579819 | 4.24E-04 | 9.65E-03 |
| hsa-miR-1260b | 2.61 | 4.21 | 0.687501 | 5.39E-04 | 1.18E-02 |
| hsa-miR-483-3p | 65.91 | 108.17 | 0.823975 | 5.91E-04 | 1.25E-02 |
| hsa-miR-625-5p | 9.7 | 6.47 | -0.579362 | 7.67E-04 | 1.56E-02 |
| hsa-miR-519a-3p | 0.8 | 1.6 | 1.04236 | 1.01E-03 | 1.99E-02 |
| hsa-miR-1307-3p | 22.83 | 33.44 | 0.547396 | 1.06E-03 | 2.00E-02 |
| hsa-miR-517c-3p | 4.09 | 7.21 | 0.806602 | 1.10E-03 | 2.02E-02 |
| hsa-miR-519a-5p | 1.02 | 1.81 | 1.00027 | 1.18E-03 | 2.10E-02 |
| hsa-miR-193a-5p | 9.17 | 14 | 0.593181 | 1.45E-03 | 2.42E-02 |
| hsa-miR-519c-3p | 1.8 | 2.97 | 0.820659 | 1.46E-03 | 2.42E-02 |
| hsa-miR-517b-3p | 10.66 | 15.79 | 0.692284 | 1.49E-03 | 2.42E-02 |
| hsa-miR-518b | 14.58 | 21.42 | 0.638291 | 1.56E-03 | 2.46E-02 |
| hsa-miR-512-3p | 1.19 | 2.07 | 1.08242 | 1.67E-03 | 2.50E-02 |
| hsa-miR-542-3p | 28.1 | 41.46 | 1.01287 | 1.67E-03 | 2.50E-02 |
| hsa-miR-323a-3p | 5.41 | 7.98 | 0.54563 | 1.74E-03 | 2.54E-02 |
| hsa-miR-29b-1-5p | 6.92 | 4.21 | -0.730275 | 1.84E-03 | 2.60E-02 |
| hsa-miR-382-3p | 3.95 | 6.24 | 0.648349 | 1.87E-03 | 2.60E-02 |
| hsa-miR-155-5p | 37.34 | 59.7 | 0.66028 | 1.94E-03 | 2.63E-02 |
| hsa-miR-181d-5p | 2.36 | 3.77 | 0.663341 | 2.15E-03 | 2.81E-02 |
| hsa-miR-153-3p | 1.05 | 1.87 | 0.818534 | 2.17E-03 | 2.81E-02 |
| hsa-miR-874-3p | 4.85 | 7.26 | 0.570667 | 2.85E-03 | 3.60E-02 |
| hsa-miR-708-5p | 23.91 | 33.64 | 0.473703 | 3.19E-03 | 3.95E-02 |
| hsa-miR-885-5p | 67.42 | 47.02 | -0.510904 | 3.28E-03 | 3.97E-02 |
| hsa-miR-1260a | 2.79 | 4.25 | 0.607087 | 3.71E-03 | 4.39E-02 |
| hsa-miR-190b | 98.14 | 63.31 | -0.621858 | 3.88E-03 | 4.44E-02 |
| hsa-miR-3173-5p | 3.86 | 2.39 | -0.664369 | 3.90E-03 | 4.44E-02 |
| hsa-miR-376b-3p | 1.74 | 2.9 | 0.748232 | 4.09E-03 | 4.57E-02 |
| hsa-miR-517a-3p | 14.63 | 21.4 | 0.69536 | 4.18E-03 | 4.57E-02 |
| hsa-let-7d-3p | 99.69 | 146.55 | 0.585396 | 4.39E-03 | 4.71E-02 |
| hsa-miR-4659a-3p | 1.26 | 0.67 | -0.992895 | 4.62E-03 | 4.85E-02 |
| hsa-miR-214-3p | 35.26 | 50.21 | 0.488742 | 4.69E-03 | 4.85E-02 |
| hsa-miR-1255a | 3.8 | 2.46 | -0.594781 | 4.84E-03 | 4.90E-02 |
| hsa-miR-1185-1-3p | 1.61 | 2.53 | 0.650379 | 4.90E-03 | 4.90E-02 |
| hsa-miR-5683 | 2.23 | 3.67 | 1.06517 | 5.08E-03 | 4.91E-02 |
| hsa-miR-95-5p | 8.71 | 12.9 | 0.58472 | 5.09E-03 | 4.91E-02 |
| hsa-miR-518c-3p | 3.99 | 6.39 | 0.628398 | 6.32E-03 | 5.99E-02 |
| hsa-miR-302a-5p | 2.34 | 3.59 | 0.620138 | 6.89E-03 | 6.43E-02 |
| hsa-miR-33a-5p | 2.76 | 4.36 | 0.715253 | 7.94E-03 | 7.29E-02 |
| hsa-miR-181c-3p | 1.55 | 2.4 | 0.647101 | 8.50E-03 | 7.68E-02 |
| hsa-let-7f-2-3p | 3.26 | 4.93 | 0.622827 | 8.73E-03 | 7.76E-02 |
| hsa-miR-498 | 1.23 | 1.94 | 0.721238 | 8.93E-03 | 7.82E-02 |
| hsa-miR-143-5p | 80.04 | 115.35 | 0.540096 | 9.25E-03 | 7.97E-02 |
| hsa-miR-221-5p | 4.37 | 6.35 | 0.511967 | 1.00E-02 | 8.50E-02 |
| hsa-miR-331-5p | 1.94 | 1.21 | -0.676785 | 1.07E-02 | 8.96E-02 |
| hsa-miR-1185-5p | 3.13 | 4.58 | 0.538065 | 1.10E-02 | 9.07E-02 |
| hsa-miR-3157-5p | 3.32 | 2.29 | -0.527008 | 1.15E-02 | 9.33E-02 |
| hsa-miR-374a-3p | 59.09 | 82.55 | 0.567168 | 1.20E-02 | 9.37E-02 |
| hsa-miR-526b-5p | 4.24 | 6.06 | 0.595072 | 1.21E-02 | 9.37E-02 |
| hsa-miR-135a-5p | 93.88 | 61.92 | -0.519128 | 1.21E-02 | 9.37E-02 |
| hsa-miR-409-5p | 5.64 | 7.95 | 0.481782 | 1.22E-02 | 9.37E-02 |
| hsa-miR-1323 | 3.37 | 4.93 | 0.628808 | 1.24E-02 | 9.37E-02 |
| hsa-miR-4286 | 0.74 | 1.26 | 0.740788 | 1.52E-02 | 1.14E-01 |
| hsa-miR-369-5p | 24.63 | 32.87 | 0.404737 | 1.58E-02 | 1.17E-01 |
| hsa-miR-520a-5p | 1.32 | 2 | 0.726934 | 1.70E-02 | 1.24E-01 |
| hsa-miR-139-3p | 11.11 | 14.23 | 0.381356 | 1.75E-02 | 1.26E-01 |
| hsa-miR-3591-5p | 64.03 | 48.2 | -0.473159 | 1.90E-02 | 1.33E-01 |
| hsa-miR-132-5p | 14.98 | 19.46 | 0.388575 | 1.92E-02 | 1.33E-01 |
| hsa-miR-208a-3p | 40.25 | 53.14 | 0.448933 | 1.92E-02 | 1.33E-01 |
| hsa-miR-323b-3p | 0.68 | 1.14 | 0.791981 | 1.95E-02 | 1.34E-01 |
| hsa-miR-1291 | 5.58 | 3.96 | -0.523285 | 2.09E-02 | 1.42E-01 |
| hsa-miR-548c-5p | 2.25 | 1.53 | -0.547951 | 2.16E-02 | 1.44E-01 |
| hsa-miR-2116-5p | 0.75 | 1.27 | 0.730746 | 2.24E-02 | 1.48E-01 |
| hsa-miR-1277-3p | 1 | 1.56 | 1.04424 | 2.26E-02 | 1.48E-01 |
| hsa-miR-301a-3p | 12 | 14.86 | 0.664449 | 2.41E-02 | 1.54E-01 |
| hsa-miR-497-3p | 3.46 | 4.92 | 0.498379 | 2.41E-02 | 1.54E-01 |
| hsa-miR-520f-3p | 0.88 | 1.42 | 0.794932 | 2.57E-02 | 1.61E-01 |
| hsa-miR-675-5p | 2.73 | 3.94 | 0.618503 | 2.58E-02 | 1.61E-01 |
| hsa-miR-23b-5p | 19.66 | 14.77 | -0.389449 | 2.77E-02 | 1.71E-01 |
| hsa-miR-376b-5p | 4.25 | 5.87 | 0.447793 | 2.85E-02 | 1.75E-01 |
| hsa-miR-26b-3p | 9.25 | 6.99 | -0.391678 | 2.98E-02 | 1.80E-01 |
| hsa-miR-6883-3p | 5.06 | 3.67 | -0.445561 | 3.00E-02 | 1.80E-01 |
| hsa-miR-455-3p | 12.63 | 16.59 | 0.404673 | 3.11E-02 | 1.84E-01 |
| hsa-miR-580-3p | 1.2 | 1.92 | 0.646455 | 3.15E-02 | 1.84E-01 |
| hsa-miR-6513-3p | 1.53 | 0.98 | -0.651376 | 3.17E-02 | 1.84E-01 |
| hsa-miR-1249 | 1.68 | 2.57 | 0.57887 | 3.31E-02 | 1.90E-01 |
| hsa-miR-3180-5p | 0.72 | 1.13 | 0.650685 | 3.33E-02 | 1.90E-01 |
| hsa-miR-34b-5p | 1.08 | 1.65 | 0.595592 | 3.39E-02 | 1.91E-01 |
| hsa-miR-671-5p | 14.3 | 19.59 | 0.418601 | 3.45E-02 | 1.91E-01 |
| hsa-miR-376a-3p | 55.18 | 72.9 | 0.400165 | 3.46E-02 | 1.91E-01 |
| hsa-miR-532-3p | 43.33 | 33.99 | -0.356142 | 3.59E-02 | 1.96E-01 |
| hsa-miR-28-5p | 37.95 | 29.29 | -0.349923 | 3.82E-02 | 2.07E-01 |
| hsa-miR-101-5p | 12.59 | 9.87 | -0.360747 | 4.06E-02 | 2.18E-01 |
| hsa-miR-329-3p | 2.22 | 2.99 | 0.431394 | 4.12E-02 | 2.19E-01 |
| hsa-miR-193b-5p | 70.68 | 54.03 | -0.364199 | 4.50E-02 | 2.36E-01 |
| hsa-miR-1179 | 2.32 | 3.17 | 0.438605 | 4.56E-02 | 2.36E-01 |
| hsa-miR-3605-3p | 6.66 | 5.12 | -0.372233 | 4.56E-02 | 2.36E-01 |
| hsa-miR-409-3p | 36.33 | 45.96 | 0.34338 | 4.64E-02 | 2.37E-01 |
| hsa-miR-212-5p | 0.98 | 1.45 | 0.603927 | 4.66E-02 | 2.37E-01 |
| hsa-miR-345-5p | 37.67 | 30.28 | -0.329668 | 4.91E-02 | 2.44E-01 |
| hsa-miR-411-5p | 63.57 | 81.5 | 0.350119 | 4.92E-02 | 2.44E-01 |
| hsa-miR-1255b-5p | 1.84 | 1.07 | -0.676505 | 4.94E-02 | 2.44E-01 |
| hsa-miR-4804-5p | 1.4 | 0.94 | -0.583825 | 4.97E-02 | 2.44E-01 |
| hsa-miR-127-3p | 3.65 | 4.89 | 0.421541 | 5.04E-02 | 2.45E-01 |
| hsa-miR-4640-3p | 1.06 | 0.63 | -0.696602 | 5.42E-02 | 2.61E-01 |
| hsa-miR-548au-5p | 1.56 | 1.07 | -0.524859 | 5.55E-02 | 2.66E-01 |
| hsa-miR-16-2-3p | 20.18 | 11.54 | -0.640912 | 5.64E-02 | 2.68E-01 |
| hsa-miR-4454 | 3.67 | 4.79 | 0.369357 | 5.72E-02 | 2.69E-01 |
| hsa-miR-4705 | 2.88 | 3.97 | 0.493666 | 5.80E-02 | 2.69E-01 |
| hsa-miR-550a-3p | 2.81 | 1.77 | -0.632734 | 5.82E-02 | 2.69E-01 |
| hsa-miR-3164 | 2.33 | 1.72 | -0.446045 | 6.03E-02 | 2.77E-01 |
| **Muscle CIT T12-week vs. T0** | | | | | |
| **MicroRNAs** | **CIT_T0 (TMM)** | **CIT_T12 (TMM)** | **Log2 Fold change** | **P value** | **FDR** |
| hsa-miR-483-5p | 14.14 | 29.08 | 1.062 | 8.57E-08 | 4.87E-05 |
| hsa-miR-372-3p | 0.46 | 1.6 | 1.836 | 1.43E-06 | 3.43E-04 |
| hsa-miR-483-3p | 53.06 | 107.85 | 1.094 | 1.81E-06 | 3.43E-04 |
| hsa-miR-516a-5p | 13.35 | 23.24 | 1.088 | 6.96E-05 | 9.90E-03 |
| hsa-miR-675-5p | 2.23 | 4.39 | 1.039 | 1.65E-04 | 1.83E-02 |
| hsa-miR-146b-5p | 54.35 | 101.88 | 0.909 | 1.93E-04 | 1.83E-02 |
| hsa-miR-133a-3p | 247553.79 | 186742.73 | -0.393 | 5.12E-04 | 4.16E-02 |
| hsa-miR-887-3p | 1.98 | 3.83 | 0.940 | 6.19E-04 | 4.40E-02 |
| hsa-miR-518a-3p | 1.18 | 2.43 | 0.982 | 1.36E-03 | 8.60E-02 |
| hsa-miR-542-3p | 16.33 | 34.33 | 0.839 | 2.19E-03 | 1.25E-01 |
| hsa-miR-208a-5p | 4.49 | 7.35 | 0.752 | 2.57E-03 | 1.33E-01 |
| hsa-miR-29c-3p | 10696.37 | 7714.46 | -0.478 | 3.31E-03 | 1.57E-01 |
| hsa-miR-516b-5p | 5.16 | 7.76 | 0.763 | 3.81E-03 | 1.62E-01 |
| hsa-miR-517a-3p | 16.4 | 23.03 | 0.657 | 3.99E-03 | 1.62E-01 |
| hsa-miR-208a-3p | 34.9 | 59.17 | 0.939 | 5.78E-03 | 1.89E-01 |
| hsa-miR-519a-5p | 1.53 | 2.5 | 0.788 | 5.93E-03 | 1.89E-01 |
| hsa-miR-133b | 2257.38 | 1648.19 | -0.446 | 6.10E-03 | 1.89E-01 |
| hsa-miR-675-3p | 9.44 | 15.17 | 0.716 | 6.15E-03 | 1.89E-01 |
| hsa-miR-517c-3p | 5.58 | 7.89 | 0.708 | 6.30E-03 | 1.89E-01 |
| hsa-miR-885-5p | 74.98 | 43.92 | -0.642 | 6.89E-03 | 1.94E-01 |
| hsa-miR-29b-3p | 1999.38 | 1468.39 | -0.468 | 7.16E-03 | 1.94E-01 |
| hsa-miR-519a-3p | 0.99 | 1.83 | 1.079 | 7.76E-03 | 2.01E-01 |
| hsa-miR-29a-3p | 11755.67 | 9061.44 | -0.384 | 9.31E-03 | 2.24E-01 |
| hsa-miR-29c-5p | 705.62 | 498.73 | -0.501 | 9.46E-03 | 2.24E-01 |
| hsa-miR-21-5p | 5332.1 | 8378.33 | 0.569 | 1.01E-02 | 2.30E-01 |
| hsa-miR-517b-3p | 11.97 | 16.35 | 0.619 | 1.11E-02 | 2.43E-01 |
| hsa-miR-181a-3p | 9.22 | 14.85 | 0.646 | 1.15E-02 | 2.43E-01 |
| hsa-miR-450b-5p | 11.51 | 20.68 | 0.596 | 1.22E-02 | 2.47E-01 |
| hsa-miR-26a-5p | 35715.66 | 28071.16 | -0.336 | 1.26E-02 | 2.48E-01 |
| hsa-miR-22-3p | 7454.42 | 5750.97 | -0.363 | 1.32E-02 | 2.51E-01 |
| hsa-miR-101-5p | 13.99 | 9.85 | -0.525 | 1.37E-02 | 2.51E-01 |
| hsa-miR-424-5p | 46.57 | 74.62 | 0.595 | 1.52E-02 | 2.70E-01 |
| hsa-miR-4524a-3p | 3.42 | 2.33 | -0.585 | 1.66E-02 | 2.86E-01 |
| hsa-miR-450a-1-3p | 1.07 | 2.44 | 0.919 | 1.75E-02 | 2.90E-01 |
| hsa-miR-206 | 20118.68 | 16613.22 | -0.355 | 1.86E-02 | 2.90E-01 |
| hsa-miR-126-3p | 25539.46 | 31151.96 | 0.313 | 1.88E-02 | 2.90E-01 |
| hsa-miR-1283 | 2.09 | 3.35 | 0.790 | 1.88E-02 | 2.90E-01 |
| hsa-miR-512-3p | 1.35 | 2.1 | 0.762 | 1.95E-02 | 2.91E-01 |
| hsa-miR-625-5p | 10.03 | 7.2 | -0.484 | 2.00E-02 | 2.91E-01 |
| hsa-miR-144-3p | 262.25 | 368.05 | 0.768 | 2.07E-02 | 2.95E-01 |
| hsa-miR-221-5p | 4.66 | 7.03 | 0.596 | 2.23E-02 | 3.04E-01 |
| hsa-miR-3173-5p | 3.83 | 2.56 | -0.547 | 2.24E-02 | 3.04E-01 |
| hsa-miR-526b-5p | 4.35 | 5.89 | 0.487 | 2.56E-02 | 3.39E-01 |
| hsa-miR-548i | 1.07 | 1.86 | 0.856 | 2.72E-02 | 3.52E-01 |
| hsa-miR-514a-3p | 4.29 | 2.98 | -0.656 | 2.97E-02 | 3.76E-01 |
| hsa-miR-365b-3p | 964.3 | 734.71 | -0.374 | 3.33E-02 | 4.05E-01 |
| hsa-miR-365a-3p | 1068.41 | 814.7 | -0.371 | 3.34E-02 | 4.05E-01 |
| hsa-miR-579-3p | 1.51 | 0.98 | -0.753 | 3.44E-02 | 4.05E-01 |
| hsa-miR-1303 | 1.2 | 0.74 | -0.705 | 3.54E-02 | 4.05E-01 |
| hsa-miR-1307-3p | 21.98 | 29.02 | 0.408 | 3.66E-02 | 4.05E-01 |
| hsa-miR-518c-3p | 5.12 | 6.47 | 0.522 | 3.67E-02 | 4.05E-01 |
| hsa-miR-369-3p | 19.75 | 30.06 | 0.521 | 3.71E-02 | 4.05E-01 |
| hsa-miR-221-3p | 368.82 | 501.37 | 0.445 | 3.93E-02 | 4.18E-01 |
| hsa-miR-519c-3p | 2.11 | 2.86 | 0.593 | 3.97E-02 | 4.18E-01 |
| hsa-miR-497-3p | 3.57 | 4.9 | 0.569 | 4.29E-02 | 4.44E-01 |
| hsa-miR-598-3p | 10.09 | 13.7 | 0.446 | 4.58E-02 | 4.62E-01 |
| hsa-miR-363-3p | 76.38 | 94.49 | 0.580 | 4.64E-02 | 4.62E-01 |
| hsa-miR-1277-5p | 12.11 | 16.42 | 0.528 | 4.71E-02 | 4.62E-01 |
| hsa-miR-518f-3p | 7.21 | 8.62 | 0.562 | 5.32E-02 | 5.09E-01 |
| hsa-miR-299-3p | 2.01 | 3 | 0.542 | 5.36E-02 | 5.09E-01 |
| hsa-miR-302b-3p | 0.64 | 1.09 | 0.713 | 5.58E-02 | 5.20E-01 |
| hsa-miR-32-3p | 1.85 | 1.27 | -0.542 | 5.87E-02 | 5.31E-01 |
| hsa-miR-376a-5p | 2.05 | 3.18 | 0.575 | 5.88E-02 | 5.31E-01 |
| hsa-miR-431-5p | 3.47 | 5.26 | 0.516 | 6.00E-02 | 5.33E-01 |

| **Serum PLA T12-week vs. T0** | | | | | |
| --- | --- | --- | --- | --- | --- |
| **MicroRNAs** | **PLA_T0 (TMM)** | **PLA_T12 (TMM)** | **Log2 Fold change** | **P value** | **FDR** |
| hsa-miR-4433b-3p | 13.22 | 36.96 | 1.37995 | 7.43E-04 | 2.65E-01 |
| hsa-miR-206 | 45.25 | 167.24 | 1.35772 | 1.20E-03 | 2.65E-01 |
| hsa-miR-6511b-3p | 27.39 | 10.61 | -1.34634 | 1.32E-03 | 2.65E-01 |
| hsa-miR-6879-3p | 0 | 5.91 | 3.95485 | 1.97E-03 | 2.65E-01 |
| hsa-miR-16-5p | 205984.2 | 190494 | -0.152022 | 2.20E-03 | 2.65E-01 |
| hsa-miR-99a-5p | 255.96 | 143.1 | -0.723232 | 2.58E-03 | 2.65E-01 |
| hsa-miR-497-5p | 16.47 | 4.67 | -1.75577 | 2.70E-03 | 2.65E-01 |
| hsa-miR-5187-5p | 11.08 | 28.59 | 1.2413 | 3.33E-03 | 2.85E-01 |
| hsa-miR-214-3p | 11.48 | 2.47 | -2.03014 | 5.08E-03 | 3.87E-01 |
| hsa-miR-1247-5p | 22.94 | 8.68 | -1.53708 | 5.99E-03 | 4.11E-01 |
| hsa-miR-889-3p | 6.55 | 17.3 | 1.46757 | 6.70E-03 | 4.18E-01 |
| hsa-miR-4772-3p | 2.71 | 12.15 | 2.04637 | 2.18E-02 | 9.98E-01 |
| hsa-miR-10b-3p | 8.96 | 2.66 | -1.64894 | 2.28E-02 | 9.98E-01 |
| hsa-miR-625-5p | 8.45 | 18.48 | 1.01504 | 2.88E-02 | 9.98E-01 |
| hsa-miR-1249 | 27.7 | 56.24 | 0.88769 | 2.89E-02 | 9.98E-01 |
| hsa-miR-150-3p | 38.58 | 22.2 | -0.783906 | 3.06E-02 | 9.98E-01 |
| hsa-miR-590-3p | 17.15 | 7.6 | -1.03938 | 3.63E-02 | 9.98E-01 |
| hsa-miR-221-5p | 7.1 | 15.12 | 1.13679 | 3.77E-02 | 9.98E-01 |
| hsa-miR-487b-3p | 18.93 | 31.62 | 0.860625 | 4.27E-02 | 9.98E-01 |
| hsa-miR-7976 | 9.82 | 18.6 | 1.01198 | 4.65E-02 | 9.98E-01 |
| hsa-miR-885-5p | 35.29 | 22.72 | -0.734502 | 5.06E-02 | 9.98E-01 |
| hsa-miR-125b-2-3p | 8.74 | 2.28 | -1.85445 | 5.41E-02 | 9.98E-01 |
| hsa-miR-6791-3p | 5.3 | 1.23 | -1.89978 | 5.54E-02 | 9.98E-01 |
| **Serum CIT T12-week vs T0** | | | | | |
| **MicroRNAs** | **CIT_T0 (TMM)** | **CIT_T12 (TMM)** | **Log2 Fold change** | **P value** | **FDR** |
| hsa-miR-744-5p | 1896.46 | 961.74 | -0.999376 | 1.92E-05 | 1.08E-02 |
| hsa-miR-106b-5p | 5.53 | 64.18 | 3.20741 | 9.22E-05 | 2.60E-02 |
| hsa-miR-484 | 925.9 | 1609.82 | 0.782608 | 2.37E-04 | 3.84E-02 |
| hsa-miR-151a-3p | 3351.23 | 1846.92 | -0.913021 | 2.73E-04 | 3.84E-02 |
| hsa-miR-6511a-3p | 2.77 | 36.83 | 3.38396 | 3.76E-04 | 4.24E-02 |
| hsa-miR-501-5p | 0 | 21.28 | 5.04169 | 9.54E-04 | 8.95E-02 |
| hsa-miR-339-5p | 1258.67 | 721.8 | -0.920887 | 1.71E-03 | 1.11E-01 |
| hsa-miR-100-5p | 45.74 | 120.23 | 1.31214 | 1.94E-03 | 1.11E-01 |
| hsa-miR-182-5p | 799.27 | 1374.79 | 0.758977 | 1.94E-03 | 1.11E-01 |
| hsa-miR-370-3p | 149.43 | 40.19 | -1.52192 | 2.00E-03 | 1.11E-01 |
| hsa-miR-1307-3p | 2347.59 | 1400.29 | -0.72429 | 2.17E-03 | 1.11E-01 |
| hsa-miR-323b-3p | 75.09 | 23.78 | -1.69541 | 2.54E-03 | 1.19E-01 |
| hsa-miR-146a-5p | 11213.54 | 5671.28 | -0.985814 | 3.12E-03 | 1.35E-01 |
| hsa-miR-328-3p | 1705.46 | 1092.42 | -0.640296 | 4.09E-03 | 1.55E-01 |
| hsa-miR-28-3p | 769.27 | 395.9 | -0.983826 | 4.14E-03 | 1.55E-01 |
| hsa-miR-10b-3p | 15.3 | 0 | -4.62369 | 4.71E-03 | 1.60E-01 |
| hsa-miR-451a | 6053.85 | 11731.58 | 0.870426 | 4.83E-03 | 1.60E-01 |
| hsa-miR-221-3p | 2952.2 | 1859.97 | -0.692469 | 5.50E-03 | 1.69E-01 |
| hsa-miR-432-5p | 1522.16 | 639.48 | -1.06296 | 5.70E-03 | 1.69E-01 |
| hsa-miR-134-5p | 395.27 | 127.4 | -1.46935 | 6.22E-03 | 1.70E-01 |
| hsa-miR-1908-5p | 95.47 | 43.06 | -1.19495 | 6.33E-03 | 1.70E-01 |
| hsa-miR-505-5p | 76.9 | 28.05 | -1.35257 | 7.04E-03 | 1.80E-01 |
| hsa-let-7g-5p | 2975.43 | 4417.35 | 0.557813 | 7.77E-03 | 1.90E-01 |
| hsa-miR-409-3p | 1570.05 | 812.22 | -0.857294 | 8.58E-03 | 2.01E-01 |
| hsa-miR-1249 | 35.87 | 83.8 | 1.17229 | 1.04E-02 | 2.35E-01 |
| hsa-miR-423-3p | 1633.5 | 1136.2 | -0.580824 | 1.70E-02 | 3.69E-01 |
| hsa-miR-431-5p | 166.79 | 69.83 | -1.20755 | 1.88E-02 | 3.84E-01 |
| hsa-miR-518b | 0 | 9.11 | 3.85164 | 1.91E-02 | 3.84E-01 |
| hsa-miR-5189-3p | 4.72 | 25.73 | 2.77608 | 2.12E-02 | 4.09E-01 |
| hsa-miR-379-3p | 11.23 | 0 | -4.39535 | 2.23E-02 | 4.09E-01 |
| hsa-miR-136-3p | 1.57 | 15.55 | 2.96316 | 2.25E-02 | 4.09E-01 |
| hsa-miR-103a-3p | 8539.41 | 5672.07 | -0.582515 | 2.43E-02 | 4.16E-01 |
| hsa-miR-4433b-5p | 1541.01 | 920.54 | -0.644822 | 2.44E-02 | 4.16E-01 |
| hsa-miR-1273h-3p | 31.2 | 7.69 | -1.98874 | 2.79E-02 | 4.62E-01 |
| hsa-miR-4669 | 7.27 | 0 | -3.82903 | 3.04E-02 | 4.74E-01 |
| hsa-miR-6783-3p | 0 | 11.28 | 3.76375 | 3.12E-02 | 4.74E-01 |
| hsa-miR-3163 | 0 | 11.28 | 3.76375 | 3.12E-02 | 4.74E-01 |
| hsa-miR-187-3p | 12.11 | 0 | -3.72693 | 3.24E-02 | 4.80E-01 |
| hsa-miR-4646-3p | 3.04 | 16.62 | 2.29056 | 3.42E-02 | 4.88E-01 |
| hsa-miR-6859-5p | 12.65 | 0 | -3.81349 | 3.47E-02 | 4.88E-01 |
| hsa-miR-3154 | 0 | 10.72 | 3.56625 | 3.74E-02 | 4.94E-01 |
| hsa-miR-6741-3p | 24.52 | 6.26 | -1.94219 | 3.75E-02 | 4.94E-01 |
| hsa-miR-1179 | 0 | 6.26 | 3.42137 | 4.02E-02 | 4.94E-01 |
| hsa-miR-483-3p | 316.83 | 562.33 | 0.794369 | 4.07E-02 | 4.94E-01 |
| hsa-miR-584-5p | 1127.08 | 838.05 | -0.632424 | 4.33E-02 | 4.94E-01 |
| hsa-miR-766-5p | 17.64 | 1.25 | -2.53284 | 4.44E-02 | 4.94E-01 |
| hsa-miR-6721-5p | 49.66 | 15.72 | -1.45505 | 4.46E-02 | 4.94E-01 |
| hsa-miR-4742-3p | 6.08 | 31.47 | 2.12654 | 4.56E-02 | 4.94E-01 |
| hsa-miR-496 | 8.03 | 0 | -3.48704 | 4.64E-02 | 4.94E-01 |
| hsa-miR-4749-5p | 0 | 6.61 | 3.26068 | 4.89E-02 | 4.94E-01 |
| hsa-miR-101-3p | 1476.19 | 2032.18 | 0.405748 | 4.98E-02 | 4.94E-01 |
| hsa-miR-1323 | 0 | 6.25 | 3.46574 | 5.15E-02 | 4.94E-01 |
| hsa-miR-211-5p | 16.5 | 3.76 | -2.51119 | 5.22E-02 | 4.94E-01 |
| hsa-miR-543 | 7.11 | 0 | -3.44869 | 5.34E-02 | 4.94E-01 |
| hsa-miR-551a | 5.43 | 0 | -3.55431 | 5.42E-02 | 4.94E-01 |
| hsa-let-7f-2-3p | 5.43 | 0 | -3.55431 | 5.42E-02 | 4.94E-01 |
| hsa-miR-500a-5p | 6.08 | 0 | -3.49152 | 5.42E-02 | 4.94E-01 |
| hsa-miR-485-5p | 76.24 | 29.1 | -1.23996 | 5.51E-02 | 4.94E-01 |
| hsa-miR-139-5p | 73.48 | 35.9 | -0.915956 | 5.79E-02 | 4.94E-01 |
| hsa-miR-450b-5p | 10.85 | 33.41 | 1.61344 | 5.92E-02 | 4.94E-01 |
| hsa-miR-4750-3p | 0 | 8.04 | 3.21339 | 5.95E-02 | 4.94E-01 |

**Table S5**

| **microRNAs** | **T0_p** | **T12_p** | **FC** | **FDR p-value** | **NGS Comparaison** |
| --- | --- | --- | --- | --- | --- |
|  | **TMM** | **TMM** |  |  | **T0 vs T12** |
| miR-483-5p | 14.1 | 29.1 | 1.1 | 4.85E-5 | HIIT-CIT (muscle) |
|  | 16.7 | 30.2 | 0.9 | 0.0002 | HIIT-PLA (muscle) |
| miR-483-3p | 53.1 | 107.8 | 1.1 | 0.0003 | HIIT-CIT (muscle) |
|  | 65.9 | 108.2 | 0.83 | 0.012 | HIIT-PLA (muscle) |
| miR-516a-5p | 13.3 | 23.3 | 1.1 | 0.0039 | HIIT-CIT (muscle) |
|  | 8.0 | 22.0 | 1.57 | 3.78E-8 | HIIT-PLA (muscle) |
| miR-369-3p | 17.8 | 27.1 | 0.58 | 0.01 | HIIT-PLA (muscle) |
| miR-136-3p | 18.1 | 28.0 | 0.7 | 0.0072 | HIIT-PLA (muscle) |
| miR-136-5p | 11.1 | 18.5 | 0.75 | 0.007 | HIIT-PLA (muscle) |
| miR-146b-5p | 54.3 | 102.0 | 0.91 | 0.018 | HIIT-CIT (muscle) |
|  | 58.3 | 115.0 | 0.96 | 0.0015 | HIIT-PLA (muscle) |
| miR-133a-3p | 251401 | 182131 | -0.46 | 0.00E+00 | HIIT-PLA (muscle) |
|  | 247554 | 186743 | -0.40 | 0.042 | HIIT-CIT (muscle) |
| miR-504-5p | 23.3 | 37.4 | 0.74 | 0.0025 | HIIT-PLA (muscle) |
| miR-1277-5p | 10.7 | 18.9 | 1.04 | 0.007 | HIIT-PLA (muscle) |
| miR-181a-3p | 8.4 | 13.4 | 0.70 | 0.004 | HIIT-PLA (muscle) |
| miR-625-3p | 19.4 | 12.5 | -0.62 | 0.005 | HIIT-PLA (muscle) |
|  | 422.3 | 280.8 | -0.56 | 0.64 | HIIT-CIT (serum) |
|  | 421.0 | 396.0 | -0.08 | 0.99 | HIIT-PLA (serum) |
| miR-515-5p | 6.3 | 12.2 | 1 | 0.00021 | HIIT-PLA (muscle) |
| miR-127-5p | 6.6 | 11.1 | 0.74 | 0.0013 | HIIT-PLA (muscle) |
| miR-744-5p | 1896.5 | 961.7 | -0.99 | 0.011 | HIIT-CIT (serum) |
| miR-484 | 926 | 1610 | 0.78 | 0.038 | HIIT-CIT (serum) |
| miR-151a-3p | 3351 | 1847 | -0.91 | 0.04 | HIIT-CIT (serum) |
| miR-4433b-5p | 1541 | 920 | -0.64 | 0.04 | HIIT-CIT (serum) |
| miR-106b-5p | 5.5 | 64.2 | 3.21 | 0.025 | HIIT-CIT (serum) |

**Table S6 A**

| **MicroRNA**s | **Tissu expression** | **Biological process** | **Validated mRNA Targets** | **References** |
| --- | --- | --- | --- | --- |
| -133a-3p  -133b | Skeletal Muscle-specific  cardiac tissue  adipocyte  bone | Myogenesis/regeneration  Protein anabolism  Mitochondrial biogenesis  Glucose homeostasis  Insulin resistance  Satellite stem cell | IGF-1, IGF-1R, SRF, MEF2, c-Met, HGF FOXO1, LIF, HDAC-1-7, NFATc4, Col8A1, col6A3, MYH9, MYO18A, UCP2, SP1, PAX7, KLF15, RUNX2, PRMDM16, SMARCD1, AKT/mTOR/S6K | PMID: 26708096  PMID: 23395168 PMID: 16614355 PMID: 21606874 PMID: 20724363 PMID: 25553440 PMID: 30235878 |
| -206 | Skeletal Muscle-specific, higher in soleus vs plantaris | Myogenesis/regeneration  Protein anabolism  Responsive to muscle damage  Satellite stem cell specification | PaX3, Notch3, IGFBP5, Hmgb3, Pola1, Erα, Akt, RunX1, B-ind1, Cx43, Mmd, c-Met, Ftl1, Mef2a, Hdac4 | PMID: 26708096  PMID: 20724363 PMID: 25553440 |
| -208b | Cardiac tissue, muscle | Muscle fiber shift  Promotes muscle growth | P70S6K, CBx1, MED13, MSTN, PURB, SOX6, SP3 | PMID: 19922871 PMID: 26708096  PMID: 19828686  PMID: 25553440 |
| -499-5p | Ubiquitous muscle | Muscle fiber shift  Promotes muscle growth | CBx1, MAPK6, NED13, MSTN, PURB, SOX6, SP3 | PMID: 19922871  PMID: 19828686  PMID: 25553440 |
| -151a-3p | Vessel wall, heart, skeletal muscle, bone marrow, lungs | Shift in muscle fiber  Decrease slow muscle gene expression  Decrease proliferation and differentiation of muscle cells | MHC-b/slow, MHC-2a, ATP2a2, SERCA2, RALGAPA1, TnI-S,  DCN | PMID: 25200835 |
| -504-5p | Vascular wall | Regulates negatively protein anabolism and insulin sensitivity | GRb10, EgR2, IGF2R, TP53 | PMID : 27994142  PMID ; 26941017 |
| -136-3p | Ubiquitous, vascular endothelial cells, muscle | Interfers with myocyte glucose uptake | DLK1-DTO3, Notch3, MTDH, PTEN, IL-17, EIF5A2 | PMID: 32434208  PMID; 23154418  PMID: 32072664 |
| -483-3p | Muscle, cardiac tissue, adrenal cortex  Vascular wall, endothelial cells | Inhibits myoblast proliferation, differentiation  Protective effect on endothelial function in hypertension | IGF-1/PI3K/Akt, IGF-2,  ACE1, ET-1, CTGF, TGF-alpha | [http://multimir.org](http://multimir.org/)  PMID: 27346130 |
| -484 | Cardiac tissue | Regulates mitochondrial fission and apoptosis | TNF-alpha, IL-6, IL-1, YAP1, Fis1, SMAD7 | PMID: 35356223  PMID: 22510686 |
| -744-5p | Muscle, pancreas | Negative regulators of myogenesis  Inhibits myoblast differentiation into myotubule | Wnt5a/Ca++ CaMKIIdelta, SIRT6 | PMID : 31051333  PMID : 34663177 |
| -106b-5p | Ubiquitous, skeletal muscle, heart, adipocyte | Interfers with glucose uptake, transport and satellite stem cell function and muscle regeneration | UCP1, (SLC2A4, GLUT4), PTEN, Akt-mTORC1*, IGF-1R | PMID: 27165190  PMID: 23954633  PMID: 28428964 |
| -4433b-5p | Extracellular vesicles in body fluids |  | IGF-1/IGF-1R signaling pathway: IGF-1R (insulin growth factor 1 receptor) IGF-1R, IGFL2, IGF2BP1-5, FOXC1 | [http://multimir.org](http://multimir.org/)  Nucleic Acids Research, 2014, Vol. 42, No. 17 |
| -146b-5p | Skeletal muscle, heart, smooth muscle cells | Regulate myoblast proliferation, differentiation, osteogenic differentiation | Smad4, NF-kB/TNFalpha signaling | PMID: 24956113 |
| -127-5p | Cartilage, muscle, liver | Suppress Muscle Mitochondrial ATP Synthase β | Beta-F1-ATPase, osteopontin | PMID: 22433606 |

**Table S6 B**

| **microRNA** | **Target Sequence and Paired microRNA** | **Site Type** | **Context Score** | **Context Score Percentile** | **Weighted Context Score** | **Conserved Branch Length** | **Pct** |
| --- | --- | --- | --- | --- | --- | --- | --- |
| hsa-miR-4433b-5p | Position 5167-5213 of IGF-1R 3’UTR | N/A | NA | 97 | N/A | N/A | N/A |
| hsa-miR-4433b-5p | Position 2572-2578 of IGF-1R 3' UTR | 7mer-m8 | -0.08 | 61 | -0.06 | 0 | N/A |
| hsa-miR-4433b-5p | Position 1120-1127 of PIK3R2 3' UTR | 8mer | -0.25 | 91 | -0.25 | 0 | N/A |
| hsa-miR-4433b-5p | Position 3325-3331 of PIK3R3 3' UTR | 7mer-m8 | -0.14 | 75 | -0.10 | 0 | N/A |
| hsa-miR-4433b-5p | Position 393-315 of PIK3R1 3' UTR | 7mer-A1 | -0.21 | 87 | -0.21 | 0 | N/A |
| hsa-miR-4433b-5p | Position 3576-3583 of PIK3R1 3' UTR | 7mer-m8 | -0.17 | 82 | -0.16 | 0 | N/A |
| hsa-miR-483-3p | Position 170-177 of IGF-1 3' UTR | 8mer | -0.40 | 99 | -0.40 | 2.532 | N/A |
| hsa-miR-106b-5p | Position 1458-1465 of IGFBP1 3' UTR | 8mer | -0.12 | 90 | -0.10 | 3.045 | 0.59 |
| hsa-miR-106b-5p | Position 242-248 of IGFBP1 3' UTR | 7mer-m8 | -0.10 | 88 | -0.10 | 0.159 | <0.1 |

**Table S7A : Muscle**


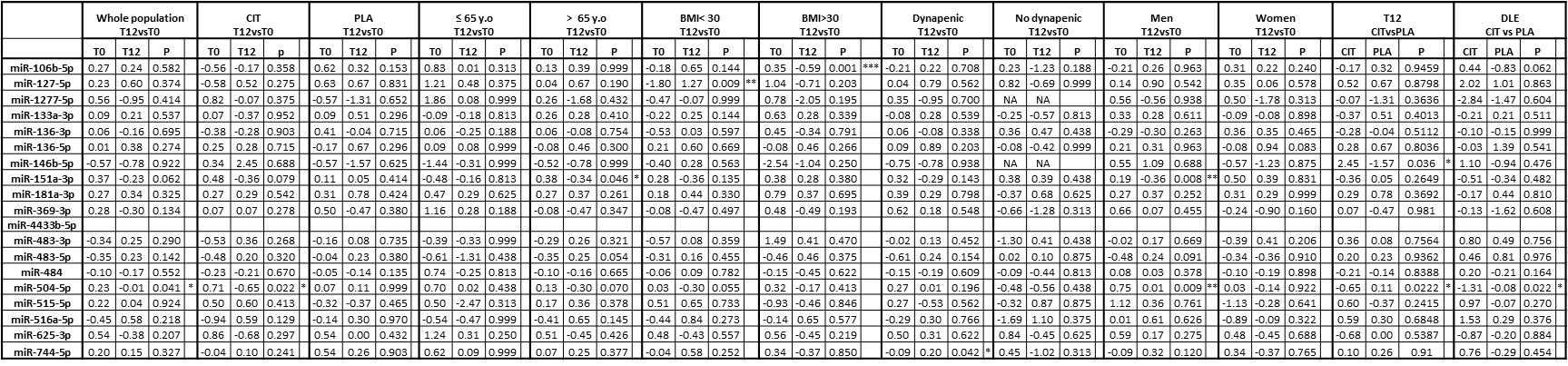


**Table S7B : Serum**

**
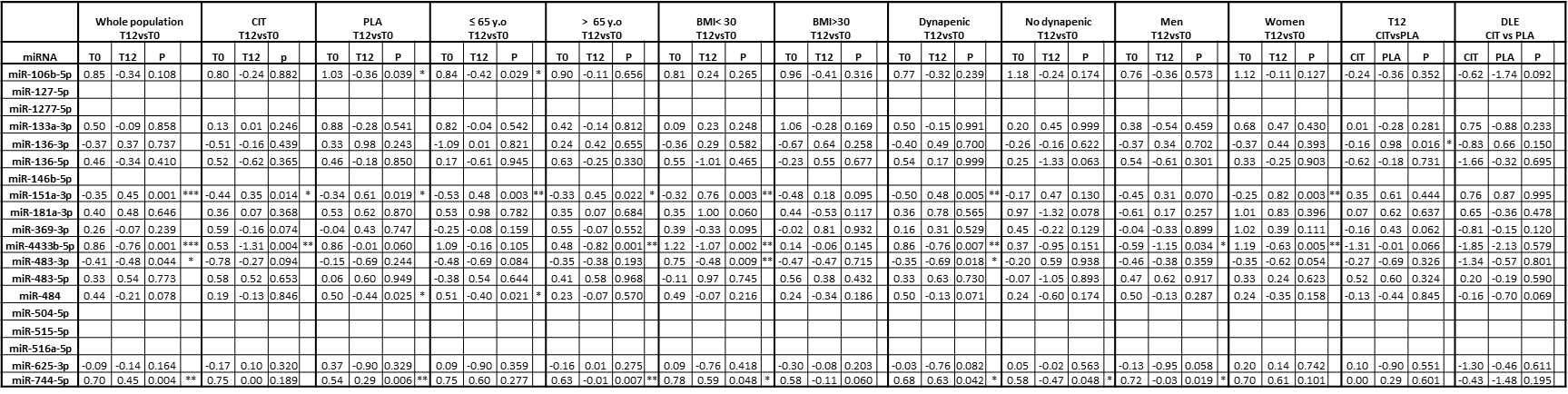
**

**Supplementary Information**

1. *Inclusion and exclusion criteria*

To be included in this study, participants had to meet the following criteria:

1. Autonomous (being able to follow the exercise program),
2. Obesity (BMI between 30 and 40 kg / m2) or a waist circumference greater than 102 cm for men and 88 cm for women or fat mass (%; total or androïd or gynoïd) equal or superior to 27% in men and 40% in women;
3. Stable weight (± 5 kg) for 6 months;
4. Non-smokers and moderate drinkers (max: 15 g/day of alcohol);
5. No history of stroke
6. Inactive (less than 2 hours of structured physical activity per week);
7. No involvement in a vigorous exercise program for at least 12 months;
8. Ability to understand French;
9. Postmenopausal for women (over 60 consecutive months without menses).
10. Availability of a high-quality serum sample before and after the procedure

Exclusion criteria were the following:

1. Presence of metal implant (pacemaker);
2. Asthma requiring oral steroid treatment;
3. Use of medication that could affect metabolism or cardiovascular function;
4. Use of anticoagulants (only for participants undergoing biopsies);
5. Use of hormonal-replacement therapy.
6. Participants with diagnosed (untreated) neurological, cardiovascular, lung diseases or cognitive disorders were excluded
7. Metabolic syndrome was defined as having at least 3 components including WC criteria; Sarcopenic status was defined using the following validated equation: appendicular (leg + arms) lean mass/height (m^2^) and these criteria for women: <5.5 kg/m^2^ and for men: <7.7 kg/m^2^; Dynapenic status was defined using the following validated equation: HS/BW (kg/kg) and these criteria for women: < 0.35kg/kg and for men: < 0.42kg/kg. BMI obesity criteria was defined as having a BMI > or equal to 30 kg/m2; WC/HC (waist/hip circumference) ratio metabolic criteria was defined as having a ratio > or equal to 1.

**Supplementary Methods**

Statistical analysis of clinical data

Quantitative results are expressed as mean ± SD. The Levene’s test was used to assess the homogeneity of variances. A linear mixed-models approach (R-package nlme ) with a two-factor repeated measures ANOVA was then used to test the intervention effect (Time effect: T0 and T12), the supplementation effect (Supp- effect: PLA and CIT) and their interaction (Time×Supp effect) on the outcomes. Results were considered statistically significant when p-value < 0.05. These statistical analyses were performed using the software R (3.6.2) (foundation for statistical computing, Vienna, Austria).

Discovery phase: miRNome analysis by Next Generation Sequencing

*Small RNA isolation from muscle biopsy:* Total RNA was extracted from 50 mg per muscle biopsies in average (n=13) with the miRNeasy Mini Kit (QIAGEN®) according to manufacturer recommendations. Frozen muscle biopsy was crushed in powder using a pestle mortar placed in liquid nitrogen and homogenized in Qiazol (700 μl). Samples were vortexed vigorously after addition of chloroform (140 μl) and centrifuged for 3 min. at 12 000g. Total RNA was precipitated by mixing the supernatant with 70% ethanol (1V) and purified on a RNeasy spin column. After washings (buffer RW1, RPE), total RNA was eluted by RNAse/DNAse-free H2O and stored at -80°C.

*Small RNA isolation from serum:* Total RNA was extracted from 400 μl serum (n=9) according to manufacturer’s protocol. Samples were thawed on ice and incubated with Qiazol lysis buffer (5vol) for 5 min prior to chloroform addition (400μl) and centrifugation for 15 min, 12000g at +4°. The upper aqueous phase was incubated with 100% ethanol (1.5 vol) and purified on a RNeasy MinElute spin column. After washings (RW1, RPE buffer and 80% ethanol) genomic material was eluted from the column with 50 μl RNAse/DNAse-free H2O and stored at -80°C.

*Next Generation sequencing:* MicroRNAs expression in muscle biopsy and serum was performed by NGS analysis after total RNA extraction, library preparation and quantification followed by microRNA sequencing (Illumina platform, QIAGEN® Genomic Services). Total RNA extracted from 400 µl of serum with the microRNAeasy/plasma (QIAGEN®) and from 50 mg of muscle biopsy as described above was converted into microRNA NGS libraries using the QIAseq microRNA library kit (QIAGEN®). Adapters containing UMI (Unique Molecular Index) were ligated to the 3’-OH and 5’-P RNA prior to the reverse-transcriptase reaction to generate cDNA. The cDNA was purified with QMN beads after a 22 cycle PCR amplification with primers containing sample specific indexes allowing the library identification in the sequencing analysis. A fraction corresponding to the microRNA size range was isolated by gel electrophoresis and analyzed on the Bioanalyser 2100 (Agilent) to perform quality control (QC). After a final quantification by qPCR, the libraries were pooled in equimolar and optimal concentrations to generate the clusters on the flow cell surface before sequencing on a NextSeq500 sequencing instrument. The raw data were de-multiplexed, corrected for amplification bias and reads were aligned to microRNA sequences known in miRBase21 and on the reference genome GRCh37. Measurements were expressed as counts and normalized by dividing the count for each microRNA by the total count in a sample multiplied by 10^6^. The microRNA level was further normalized by the Trimmed Mean of M-values (TMM) method and compared between groups by the TMM ratio expressed as relative data as Log_2_ (Fold change) (Log_2_FC).

*Validation study:* MicroRNAs by Real-Time Quantitative Polymerase Chain Reaction

MicroRNAs were quantified on total RNA extracted from muscle biopsy (n=28) and serum (n=68) by TaqMan Advanced microRNA technology (Applied Biosystems, ThermoFisher Scientific. The cDNA was synthesized from 3.7 μl of 40ng/μl of total RNA for muscle biopsy and from 2 μl total RNA for serum, using a TaqMan Advanced microRNA cDNA synthesis kit. Quantification of microRNAs expressed at low copy number was improved by 14 cycles of cDNA amplification in a 2X TaqMan PreAmp master mix containing Megaplex PreAmp primers. The qPCR amplification was performed on 1:20 dilution of cDNA obtained by miR-Amp reaction on 5 μl of the RT reaction, using the 2X Fast Advanced Master Mix and the 20X TaqMan Advanced microRNA Assays (Table S2). MicroRNAs were quantified using TaqMan array card (Applied Biosystems, Thermofisher®). Amplified cDNAs (20µl) were mixed with TaqMan Fast Advanced Mastermix and 100 µl of each sample were added to the array tanks. The TaqMan array microRNA cards were designed for the quantification in duplicate of 23 microRNAs (19 candidate microRNAs, 3 endogenous controls, miR-191-5p, miR-222-3p and miR-361-5p and the exogenous control) by real-time PCR reaction on a QuantStudio® 7 flex (Applied Biosystems) according to the manufacturer’s protocol (Table S2). The C_T_ (threshold cycle value) was recorded as the cycle number at which the fluorescence generated within a reaction crosses the fluorescence threshold, a fluorescent signal significantly above the ROX™ fluorescence background recorded in each sample. We used the software Expression Suite (Applied Biosystems) to express the microRNA level as relative quantification (RQ). The Ct values of each microRNA were normalized with the mean of expression level of three endogenous controls. RQ was calculated as 2^–ΔΔCT^, with ΔC_T_ = (C_T_ microRNA – C_T_ mean of endogenous controls) and ΔΔC_T_ = (ΔC_T_ of the microRNA –ΔC_T_ mean of the microRNAs through all samples) and converted as FC = Log_2_(2^–ΔΔCT^).

**Supplementary Results**

*NGS data set analysis*:The average number of reads was 14 million reads/per sample, with an average of 1.3 (serum) and 2.5 million (muscle biopsy) UMI (Unique Molecular Index)-corrected reads for each sample. The read length (single-end read: up to 46 bp insert+19 bp 3’linker+10 UMIs) given by the number of reads was 75 nucleotides (nt). The trimming of adapters creates a read length distribution of sequences with different lengths. See below in 2 representative samples for muscle biopsy and serum: the reads representing microRNAs have a length of ~18-22 nt, longer sequences of other origin have a length of ~30-50 nt (rRNA, tRNA, mRNA, and Y-RNA fragments).

Muscle biopsy:


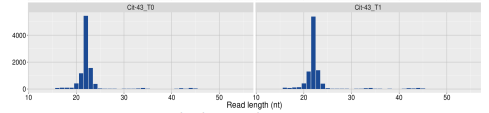


Serum sample:


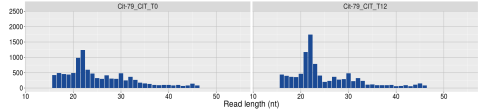


The sample QC (quality control) of analysis, given by the Q-score showed that all samples had Q-score above 30. This indicated an error probability for incorrect base call of 0.001 and assessed for high average read quality of the UMI-corrected reads.

The QC of NGS data sets also included:

-The Cp ratio of the miR-451a vs the stable hsa-miR-23a measured RT-qPCR in serum samples, with a ratio above 6, indicator of hemolysis.


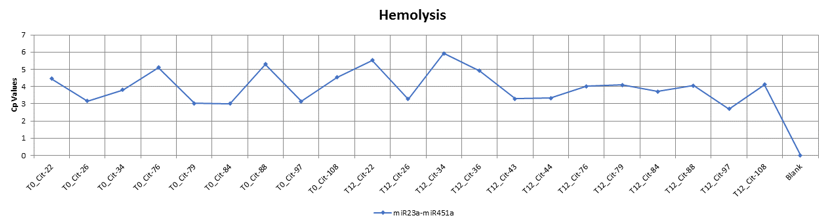


-The absence of PCR inhibitors, assessed by the RT-qPCR efficiency after running the analysis on each sample using the UniSp6, 100, 101 primer Spike-In assays (miRCURY LNA™ Universal RT microRNA PCR, Exiqon), cp: crossing point.


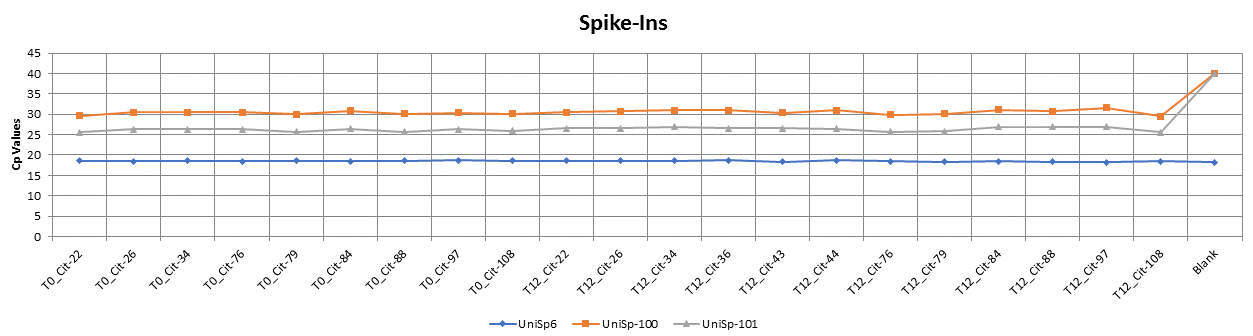


Each point on the x-axis indicates a serum sample with the last point being experimental controls. Cp: crossing point.

-The C_p_ values of endogenous microRNAs which are usually stably expressed in serum samples.

(hsa-miR-103a-3p, hsa-miR-191-5p, hsa-miR-451a, hsa-miR-23a-3p and hsa-miR-30c-5p) were analyzed by RT-qPCR. A C_T_ difference within serum would indicate flaws during blood sampling, serum handling and conservation plus experimental flaws in extraction and RT handling, prior to cDNA.


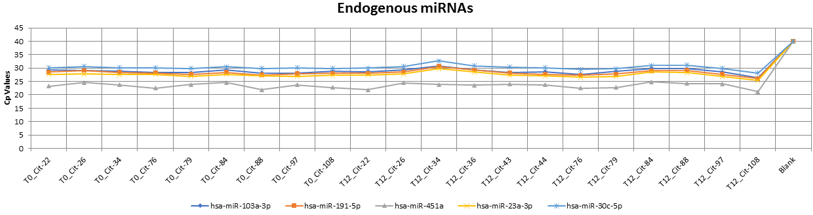


Each point on the x-axis indicates a serum sample with the last point being experimental controls. Cp: crossing point.

*Validated target genes of differentially expressed microRNAs*: To decipher the microRNA-mediated regulations of signaling pathways involved in response to a 12-weeks intervention, we searched for potential interactions of these microRNAs with targeted mRNAs (Table S6A-B).

Beside miR-133a-3p, a myo-MicroRNA targeting IGF-1/IGF-1-R, 3 non-myoMicroRNAs (miR-483-3p, 106b-5p, -4433b-5p) impact IGF-1/IGF-1-R/-R2 and AKT/mTOR/S6k signaling involved in protein anabolism (mTORC1), breakdown (FOXO1) and in mitochondrial biogenesis. More specifically, miR-151a-3p targets SERCA2, a gene encoding ATPase2, a calcium pump that downregulates slow muscle genes [34]. MiR-504-5p targets the signaling adaptator Grb10 and transcription factor Egr2 that in turn alters growth factor signaling, inflammation and cardiovascular functions [30,31]. MiR-106b-5p, a member of miR-106b-25 cluster that directly targets slc2a4 encoding glucose transporter4 (GLUT4) and its downstream signaling contributes to glucose metabolism disorder thereby to insulin resistance [46]. MiR-106b-5p decreases mRNA level of Ucp1l, a selective hall mark of brown adipocytes, and targets MYF5 in satellite muscle cells thus impairing proper muscle repair [44]. The Wnt5a and CaMKIId 3’UTRs contain several target sites of miR-744-5p and these two genes have been demonstrated as experimental targets of miR-744-5p (Peng S, et al, Mol Ther Nucleic Acids. 2019 Jun 7;16:481-493. doi: 10.1016/j.omtn.2019.03.009. Epub 2019 Apr 9. PMID: 31051333; PMCID: PMC6495097..

Using [**http://multimir.org**](http://multimir.org/)**,** we found experimentally validated targets of miR-4433-3p among the IGF-1/IGF-1R signaling pathway: IGF-1R (insulin growth factor 1 receptor) IGF-1R, IGFL2, IGF2BP1-5 and FOXC1 [34].

**Supplementary References**

[S1] Fex A, Leduc-Gaudet JP, Filion ME, Karelis AD, Aubertin-Leheudre M. Effect of Elliptical High Intensity Interval Training on Metabolic Risk Factor in Pre- and Type 2 Diabetes Patients: A Pilot Study. J Phys Act Health. 2015 Jul;12(7):942-6. doi: 10.1123/jpah.2014-0123.3

[S2] Buckinx F, Gouspillou G, Carvalho LP, Marcangeli V, El Hajj Boutros G, Dulac M, et al. Effect of High-Intensity Interval Training Combined with L-Citrulline Supplementation on Functional Capacities and Muscle Function in Dynapenic-Obese Older Adults. J Clin Med 2018;7:561. <https://doi.org/10.3390/jcm7120561>.

[S3] Youssef L, Bourgin M, Durand S, Aprahamian F, Lefevre D, Maiuri MC, et al. Serum Metabolome Adaptations Following 12 Weeks of High-Intensity Interval Training or Moderate-Intensity Continuous Training in Obese Older Adults. Metabolites 2023;13:198. <https://doi.org/10.3390/metabo13020198>.

[S4] Garmire LX, Subramaniam S. Evaluation of normalization methods in mammalian microRNA-Seq data. RNA 2012;18:1279–88. <https://doi.org/10.1261/rna.030916.111>.

[S5] Mercier-Guery A, Millet M, Merle B, Collet C, Bagouet F, Borel O, et al. Dysregulation of Micrornas in Adult Osteogenesis Imperfecta: The Miroi Study. J Bone Miner Res 2023. https://doi.org/10.1002/jbmr.4912.

[S6] Legrand MA, Millet M, Merle B, Rousseau J-C, Hemmendinger A, Gineyts E, et al. A Signature of Circulating microRNAs Associated With Fibrous Dysplasia of Bone: the mirDys Study. J Bone Miner Res 2020;35:1881–92. https://doi.org/10.1002/jbmr.4111.

[S7] Wu Y, Zuo J, Zhang Y, Xie Y, Hu F, Chen L, et al. Identification of miR-106b-93 as a negative regulator of brown adipocyte differentiation. Biochem Biophys Res Commun 2013;438:575–80. <https://doi.org/10.1016/j.bbrc.2013.08.016>.

[S8] Nielsen S, Åkerström T, Rinnov A, Yfanti C, Scheele C, Pedersen BK, et al. The microRNA Plasma Signature in Response to Acute Aerobic Exercise and Endurance Training. PLoS ONE 2014;9:e87308. https://doi.org/10.1371/journal.pone.0087308.
